# Collagen Alpha 1(XI) Amino-Terminal Domain Modulates Type I Collagen Fibril Assembly

**DOI:** 10.1021/acs.biochem.4c00434

**Published:** 2025-01-22

**Authors:** Abu Sayeed Chowdhury, Julia Thom Oxford

**Affiliations:** † Biomolecular Sciences Graduate Program, Boise State University, 1910 University Drive, Boise, Idaho 83725, United States; ‡ Biomolecular Research Institute, Boise State University, 1910 University Drive, Boise, Idaho 83725, United States

## Abstract

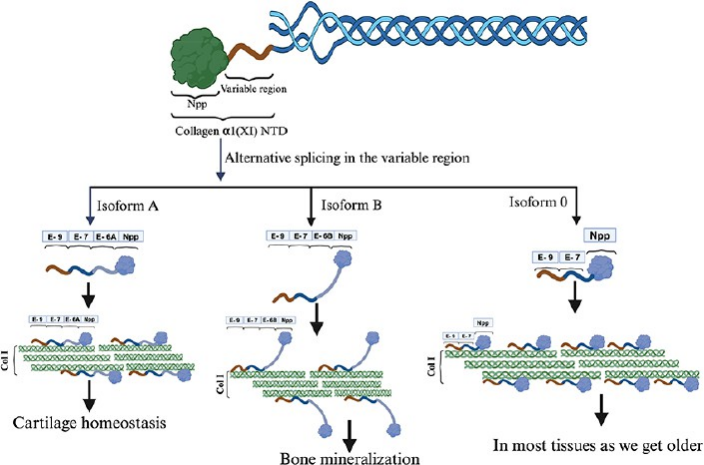

The amino-terminal domain of collagen α1(XI) plays a key role in controlling fibrillogenesis. However, the specific mechanisms through which various isoforms of collagen α1(XI) regulate this process are not fully understood. We measured the kinetics of collagen type I self-assembly in the presence of specific collagen α1(XI) isoforms. Molecular dynamics simulations, protein–protein docking studies, and molecular mechanics Poisson–Boltzmann surface area were utilized to understand the molecular mechanisms. In vitro, in silico, and thermodynamic studies demonstrated an isoform-specific effect on self-assembly kinetics. Our results indicate isoform-specific differences in the rate constants, activation energy, and free energy of binding. These differences may result from isoform-specific interaction dynamics and modulation of steric hindrance due to the chemically distinct variable regions. We show that isoform A interacts with collagen type I due in part to the acidic variable region, increasing the activation energy of fibril growth while decreasing the rate constant during the growth phase. In contrast, the basic variable region of isoform B may result in less steric hindrance than isoform A. Isoform 0 demonstrated the highest activation energy and the lowest rate constant during the growth phase. Although the presence of isoforms reduced the rate constants for fibril growth, an increase in total turbidity during the plateau phase was observed compared to controls. Overall, these results are consistent with collagen α1(XI) NTD isoforms facilitating fibrillogenesis by increasing the final yield by reducing the rate of the lag and/or growth phases, while extending the duration of the growth phase.

## Introduction

Collagen type XI is a minor fibrillar collagen that is heterotypic in nature and plays a crucial role in the nucleation, assembly, and determination of final fibril diameter.^1,2^ Fibril diameter modulation by collagen type XI may share similarities with the manner in which type V collagen controls collagen type I diameter.^2,3^ Previous studies suggest that this regulatory behavior is dependent on complex formation within the amino-terminal domain (NTD) of collagen α1(XI). It has been hypothesized that the presence of the NTD on the surface of a predominantly collagen type II fibril sterically restricts further deposition of collagen once a threshold ratio of collagen type II and collagen type XI molecules within the fibril is achieved.^4−7^

Despite being classified as minor fibrillar collagen due to its relative abundance, collagen XI is an essential component in various tissues including cartilage, bone, and muscle. Its significance becomes apparent when mutations result in congenital syndromes such as Marshall’s syndrome,^8^ Stickler’s syndrome,^9^ fibrochondrogenesis,^10^ nonsyndromic hearing loss deafness, and autosomal dominant 37.^11^ Facial and eye abnormalities, hearing loss, and articular joint abnormalities result from mutations in COL11A1, as demonstrated by these syndromic and nonsyndromic conditions.

The structure of the collagen α1(XI) NTD is composed of a commonly shared amino propeptide (Npp) encoded by exons 2–5, which is proteolytically removed during secretion, and a variable region (VR) domain, which is formed as a result of the alternative splicing of exons 6a, 6b,7, and 8.^12^ The spliced variant involving Npp, with exon combinations 6A, 7, and 9, results in the production of a highly acidic protein referred to as isoform A (Supplemental Table 1 and Supplemental Figure 1). Similarly, the spliced variant comprising Npp, exons 6B, 7, and 9 yields a highly basic protein known as isoform B (Supplemental Table 1 and Supplemental Figure 1). Additionally, there exists a distinct isoform, denoted as isoform 0, characterized by the absence of exons 6A and 6B.^13^ The splicing pattern of the variable region shows some degree of tissue-specific expression and variation; for example, isoform A is mainly present in noncartilaginous tissues as well as in cartilage, while isoform B is largely restricted to cartilage and tendon.^14,15^

Isoform B is postulated to be expressed prominently on the surface of collagen type I fibrils, as indicated by a previous study.^16^ Notably, the basic variable region of isoform B is suggested to exhibit affinity toward bone sialoprotein (BSP). The proposition is supported by coimmunoprecipitation of a 60 kDa collagen type XI amino-terminal domain (NTD) fragment, characterized by lysine triplet repeat sequences, with BSP only from mineralized osteoblastic cultures.^17^ This finding underscores the potential role of isoform B in mediating interactions crucial to the biomineralization process. These insights contribute to our understanding of the intricate molecular dynamics within osteoblastic environments and require further investigations to understand their implications in bone physiology.

**Figure 1:**
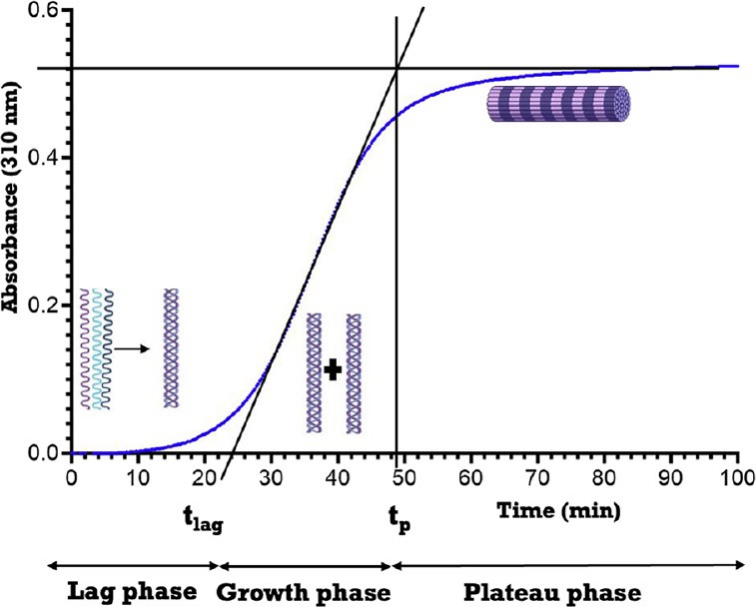
A typical turbidity–time curve depicting three phases of fibril formation. The turbidity–time curve shows the lag phase, the growth phase, and the plateau phase during collagen fibrillogenesis.

The alternative splicing of collagen α1(XI) is developmentally regulated and, due to the highly charged composition of the variable regions, may modulate biological activity, such as delaying or decreasing the rate of proteolytic processing of the Npp and, hence, the resulting fibril morphology. Previous turbidity–time assay and X-ray diffraction studies showed that in vitro collagen fibrillogenesis is initiated by the formation of linear dimers and trimers during the lag phase (Figure 1). Once the trimers are formed, rapid lateral aggregation or the growth phase begins.^18−20^ Different isoforms of collagen α1(XI) NTD may modulate the lag and growth phase kinetics based on the differential chemical nature of the amino acid residues in the variable region. To understand the self-assembly kinetics of the fibril formation of collagen type I in the presence of different isoforms of collagen α1(XI) NTD, the Arrhenius relationship can be explored. Additionally, the rate constants derived from the Arrhenius relationship can be utilized to calculate the consequential thermodynamic parameters.

The Arrhenius relationship is as follows:
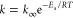
1

Here, *T* is the absolute temperature in K, *R* is the gas constant, *k*_∞_ is the rate constant as 1/*T* approaches 0, and *E*_a_ is the activation energy. The slope of the plot ln(*k*) versus 1/*T* is equal to the activation energy times over the negative gas constant. However, if we compare the rate constants at two different temperatures, the following equation of activation energy (*E*_a_) can be derived:
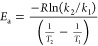
2

**Figure 2:**
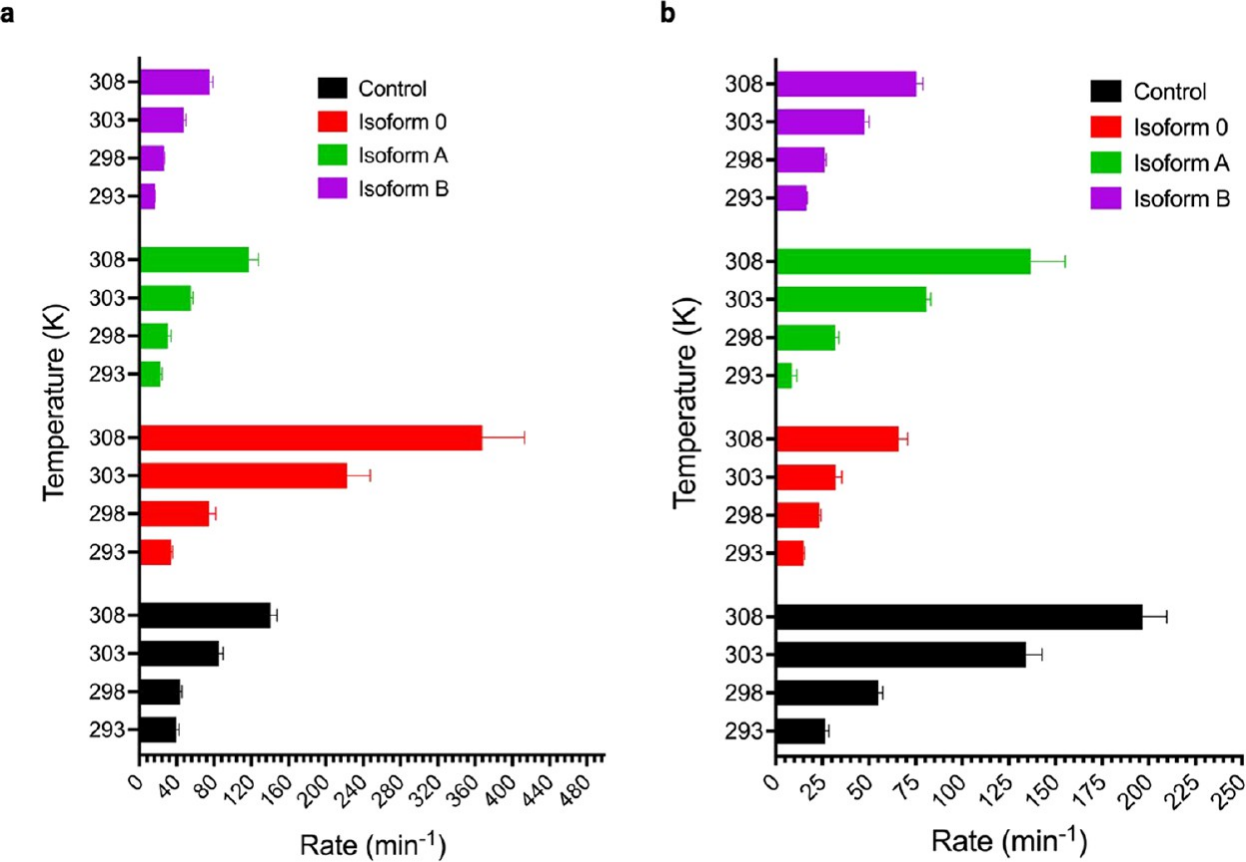
Effect of collagen α1(XI) NTD isoforms on the rate constants for collagen type I fibril formation during the lag phase and the growth phase. (a) Rate constants for fibrillogenesis are shown in the presence of isoforms A, B, and 0 compared to controls at 293, 298, 303, and 308 K during the lag phase. (b) Rate constants are shown in the presence of isoforms A, B, and 0 at 293, 298, 303, and 308 K during the growth phase. The presence of isoform 0 increased the rate constant during the lag phase at 303 and 308 K compared to the control, facilitating the formation of triple helical collagen, while the presence of isoforms A and B decreased the rate constant for the lag phase. The presence of isoforms A, B, and 0 decreased the rate constant for the growth phase compared to control. Control (black), isoform 0 (orange), isoform A (green), and isoform B (purple). Error bars represent standard deviation.

Using eq 2, we obtain the activation energy (*E*_a_) for both the lag phase and growth phase. Using the turbidity–time curve, the intersection of the tangent to the midpoint of the growth phase with the *x*-axis yields the lag time (*t*_lag_), and the plateau time (*t*_p_) is determined as the intersection between the tangent to the midpoint of the growth phase, with the horizontal line representing the plateau phase of the curve. The phase between the lag time (*t*_lag_) and plateau time (*t*_p_) represents the growth phase (Figure 1). Apparent rate constants for the lag phase and growth phase were determined from three experimental replicates by calculating *c*/*t*_lag_ × 10^3^ (mg/mL min^–1^) and *c*/ (*t*_p_ – *t*_lag_) × 10^3^ (mg/mL min^–1^), respectively, at four different concentrations and four different temperatures (Supplemental Figures 10–25). Rate constants were determined from the slope of the apparent rate versus concentration plots for specific isoforms at specific temperatures (Supplemental Figures 26–33).^21^ The mean rate constants are shown in Figure 2 for each isoform and for each temperature. Error bars represent standard deviation.

Moreover, molecular dynamics (MD) simulation and protein–protein docking studies between collagen type I and collagen α1(XI) NTD isoforms have been performed to provide insights into the molecular interactions that may occur during fibrillogenesis.

## Materials and Method

Cloning, expression, and purification of rat collagen α1(XI) NTD (isoforms A, B, and 0) were performed as per the previously published method.^22^ Using PD-10 columns from GE Healthcare, the buffer solution was exchanged with 1X PBS buffer, pH 7.4. Recombinant rat collagen α1(XI) NTD concentration was determined by using the Pierce BCA Protein Assay Kit. Rat tail collagen type I was used from Advanced Biomatrix RatCol, and the pH was adjusted to 7.4 by diluting with 1X PBS buffer. Evaluation of the impact of the isoforms A, B, and 0 of collagen α1(XI) NTD on the kinetics of self-assembly of collagen type I was performed using a turbidity–time assay. To calculate the rate constant of the fibril formation with and without the presence of collagen α1(XI) NTD isoforms, three sets of experiments were conducted. The first set of experiments was conducted with collagen type I at concentrations of 0.05, 0.1, 0.15, and 0.2 mg/mL at temperatures of 20, 25, 30, and 35 °C (293, 298, 303, and 308 K, respectively). The results of this set of experiments were used as a control. The second set of experiments was conducted using collagen α1(XI) NTD isoforms at concentrations of 0.05, 0.1, 0.15, and 0.2 mg/mL at temperatures of 20, 25, 30, and 35 °C, while maintaining a constant concentration of collagen type I at 0.15 mg/mL. These two sets of experiments were performed to calculate the rate constants in the presence and absence of collagen α1(XI) NTD isoforms. The third set of experiments was carried out with a fixed concentration of 0.15 mg/mL collagen type I, and variable concentrations of collagen α1(XI) NTD isoforms (0.05, 0.1, 0.15, and 0.2 mg/mL) were used to evaluate the aggregate/min versus concentration and total turbidity measurements at 25 °C. Mixing was carried out in ice-cold buffer placed in the cuvette of the JASCO V-750 UV–vis spectrophotometer, equipped with a temperature controller. The absorbance increase was recorded at 310 nm as a function of time. The data collection interval was 10 s with 2 nm bandwidth and 0.96 s response time. Each independent experiment was carried out in triplicate and standard deviation was calculated. Data were normalized to evaluate apparent rate constants.

### Modeling of Collagen Type I Triple Helix and Collagen α1(XI) NTD Isoforms

Two 5.18 Å rat collagen type I structures were considered from the RCSB-PDB (3HQV and 3HR2); however, these were not adequate for MD simulations. Therefore, the rat tail collagen sequence was downloaded from the UniProt database (entries: P02454 and P02466). We selected a 78 amino acid sequence from amino acids 647–724. This region included 26 Gly-Xaa-Yaa repeats. Alphafold 2 was used to build the triple helical structure of collagen type I, using two identical α1 chains and one α2 chain.^23^ Additionally, the splice variants (isoforms A, B, and 0) of collagen α1(XI) NTD were modeled using Alphafold 2, including 223 amino acids of the Npp plus 39 amino acids specific to isoform A and 51 amino acids specific to isoform B for MD simulations. The Alphafold 2-derived structure of Npp was found to be an improvement over the previously generated in silico structures in terms of structural features established in the wet lab.^24^ Two disulfide bonds between cys 146–cys 200 and cys 25–cys 207 were introduced into the Npp domain. The amino propeptide domain of collagen α1(XI) consists of 223 amino acid residues encoded by exons 2–5. Exon 6A encodes 39 amino acid residues for isoform A, rendering the protein acidic with a theoretical isoelectric point (pI) of 3.34. Conversely, exon 6B encodes 51 amino acid residues for isoform B, imparting a basic nature to the protein with a theoretical pI of 11.9.^25^

### MD Simulation

To study the interaction between the isoforms of collagen α1(XI) NTD and the triple chain of collagen type I, MD simulations were performed using the GROMACS software package with a charmm36-jul2022 force field which supports engineered amino acids.^26,27^ Parameters for MD simulations are included in Supplemental Table 6. The triple chain of collagen type I and isoforms of collagen α1(XI) were placed individually from each other at a distance ensuring that there was no initial chemical interaction using pymol software.^28^ The CHARMM-GUI server was used to transform selected prolines to hydroxyprolines and lysines to hydroxylysine as indicated in the 3HQV PDB structure and to generate the topology file (Supplemental Table 2).^29^ In the simulations, N- and C-termini as well as histidine residues were maintained in the neutral form. The proteins were maintained in a triclinic box, maintaining 1.0 nm between the protein and the edge of the box with a periodic boundary condition. After adding water molecules, the system was neutralized with CaCl_2_ to reach a physiological concentration (2.6 mmol/L). CaCl_2_ was chosen because there have been a few putative calcium-binding sites identified previously within the Npp of collagen α1(XI).^30,31^ However, to evaluate the isoform B-induced nucleation of hydroxyapatite, 0.7 M calcium ions were randomly added to enhanced sampling and accelerated simulation.^32−34^ Phosphate and hydroxyl ions were added following the stoichiometry of the hydroxyapatite crystal. The system was neutralized with sodium chloride ions. Then, energy minimization of the whole system was carried out. For the simulations, *NVT* and *NPT* ensembles were used, with a Parrinello–Rahman barostat for pressure coupling and V-rescale for temperature coupling. The electrostatic interactions were determined using the PME method, while the van der Waals interactions were calculated using a cutoff-based approach with a force-switching function and a dispersion correlation for long-range interaction. The total MD simulation extended over 100 ns at both 298 and 310 K at 1 atm pressure, with an integration step of 2 fs. The analysis of the trajectories was carried out with the GROMACS subroutines, the pymol software package, and an object-oriented Python library, MDAnalysis.^35^ To calculate the interaction-free energies in the interaction of the collagen α1(XI) NTD isoforms with collagen type I complexes, the molecular mechanics Poisson–Boltzmann surface area (MMPBSA) approach using “gmxMMPBSA” was used from the equilibrium stage extracted from the MD trajectory.^36−38^ Parameters for Gromacs molecular mechanics Poisson–Boltzmann surface area are included in Supplemental Table 7.

### Protein–Protein Docking Studies

We conducted protein–protein docking studies using HADDOCK to complement the MD simulation and to gain insights into the interactions occurring between the triple chains of collagen type I and isoforms of collagen α1(XI) NTD.^39,40^

## Results and Discussion

From the turbidity–time curves, we calculated the rate constants (Figure 2 and Supplemental Tables 3 and 4) for both the lag phase and the growth phase across specific temperatures.

**Figure 3:**
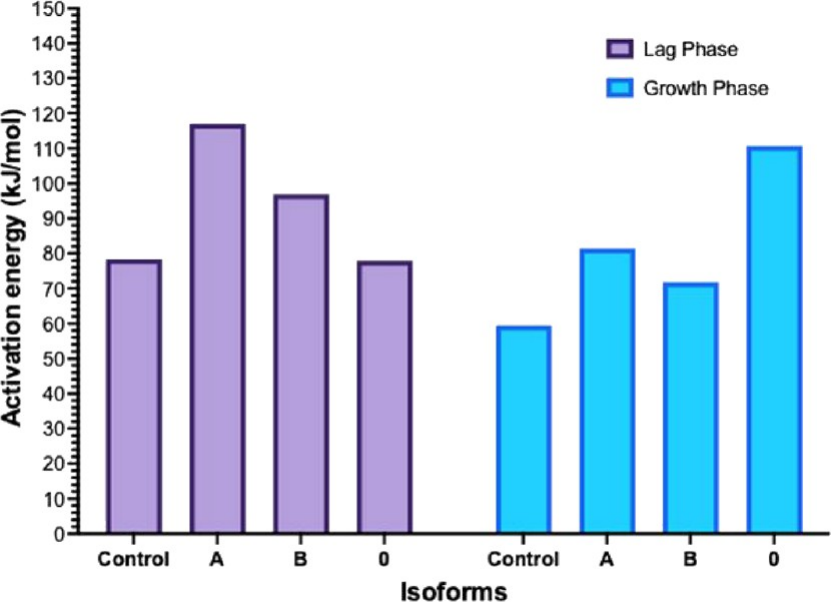
Changes in the activation energy during the lag phase and growth phase of collagen type I fibril formation due to the presence of three different isoforms of collagen α1(XI) NTD (A, B, and 0) utilizing values at 303 K (30 °C) and 308 K (35 °C). The presence of isoforms A and B increased the activation energy during the lag phase (purple bars). During the growth phase (blue bars), each isoform increased the activation energy compared to the control (collagen type I only).

While the rate constants were calculated at the specific temperatures, the activation energy was calculated by using data from two different temperatures. Using eq 2, we calculated the activation energy (*E*_a_) by employing values from 30 °C (303 K) and 35 °C (308 K) (Figure 3). We found that under conditions where the rate constant increased, the activation energy was observed to decrease in most cases. For example, during the growth phase, the addition of each isoform decreased the rate constant and increased the activation energy. During the lag phase, the addition of isoforms A and B decreased the rate constant and increased the activation energy. However, while the addition of isoform 0 increased the rate constant during the lag phase, it did not change the activation energy when compared to control (collagen type I only) (Figures 2 and 3). The observation that the activation energy was increased during the lag phase upon the addition of isoforms A and B, and increased by all during the growth phase, may result from an interaction between the isoforms of collagen α1(XI) NTD and the collagen type I that hinders triple helical collagen formation in the case of isoforms A and B but limits lateral growth by imposing steric hindrance at the surface of the growing fibril. The differences observed between the isoforms may be due to the chemically distinct variable regions, which may dictate the nature of the interaction with collagen type I.

**Figure 4:**
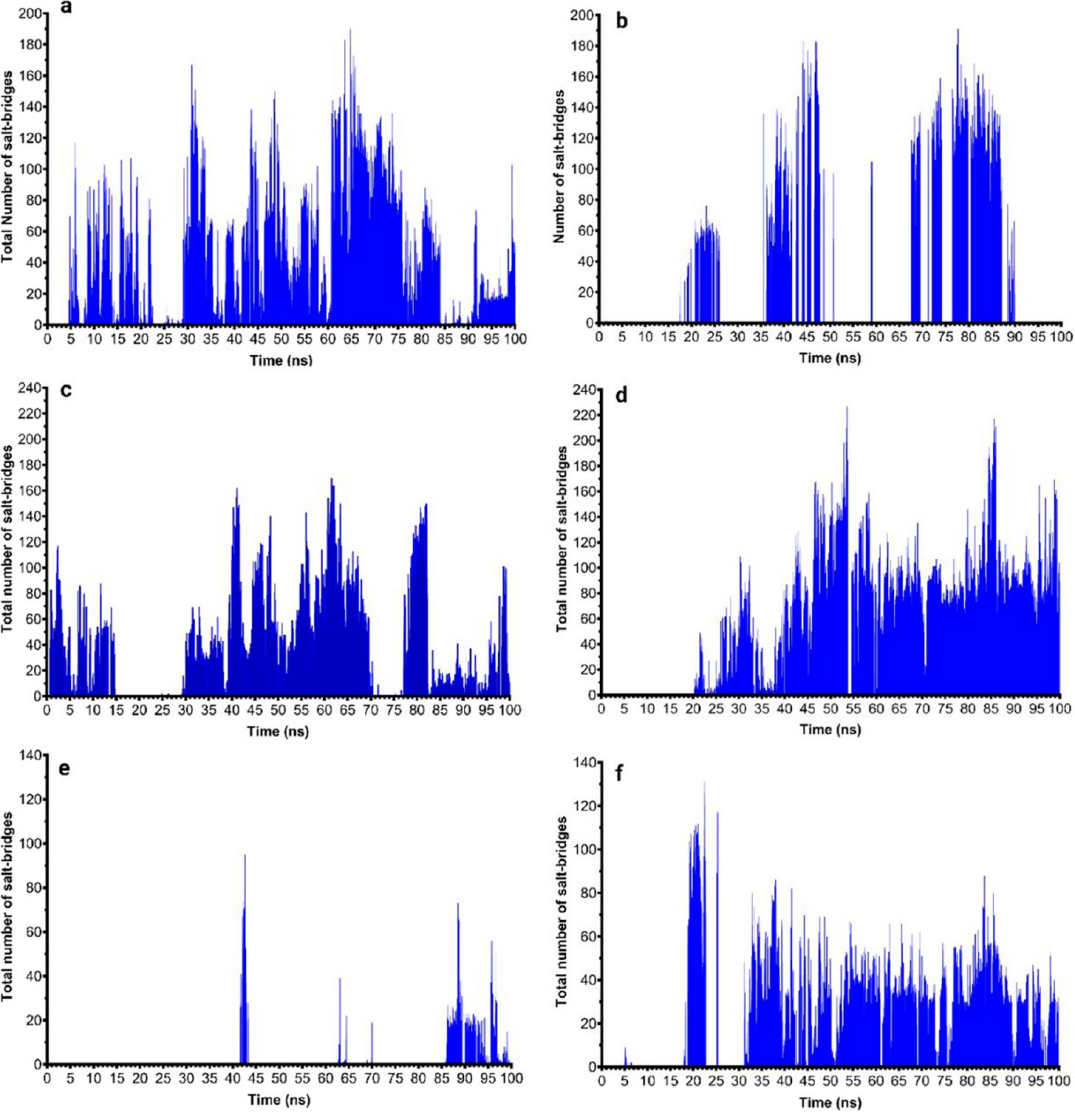
Dynamics of salt-bridge formation between collagen type I and isoforms of the collagen α1(XI) NTD: (a,b) isoform A at 37 and 25 °C, respectively; (c,d) isoform B at 37 and 25 °C, respectively; (e,f) isoform 0 at 37 and 25 °C, respectively.

**Figure 5:**
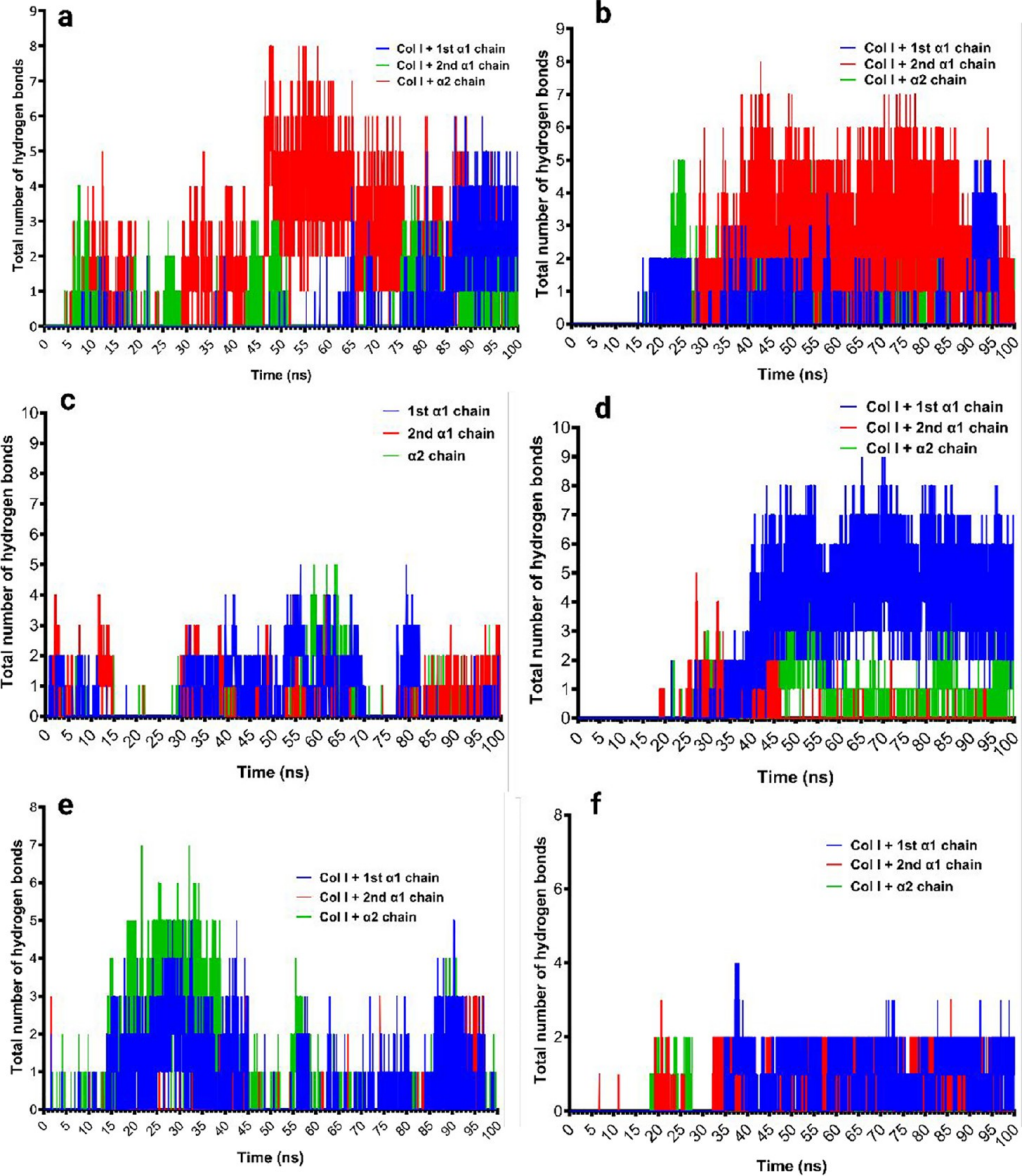
Dynamics of hydrogen bond formation between collagen type I and the isoforms of collagen α1(XI) NTD: (a,b) isoform A at 37 and 25 °C, respectively; (c,d) isoform B at 37 and 25 °C, respectively; (e,f) isoform 0 at 37 and 25 °C, respectively.

The MD simulation at two different temperatures (25 and 37 °C) suggested a temperature-dependent interaction between collagen type I and isoforms of collagen α1(XI) NTD. The center of mass (COM) distance, the total solvent accessible surface area (SASA) (Supplemental Figure 2), salt bridges (Figure 4), and hydrogen bonds (Figure 5) formation between the three chains of collagen type I and collagen α1(XI) NTD over the 100 ns simulation indicated that protein–protein interactions occur for the three isoforms.

The dynamics of interaction analysis of the collagen α1(XI) NTD isoforms and collagen type I revealed that isoform A formed the highest number of salt bridges (Figure 4a,b) and hydrogen bonds (Figure 5a,b) compared to isoforms 0 and B, whereas isoform B formed fewer salt bridges at 37 °C (Figure 4c) and hydrogen bonds (Figure 5c) at 37 °C compared to isoform A. The higher number of salt bridges at 37 °C compared to 25 °C for isoform A may indicate a more stable complex formation at physiological temperature. Such an interaction between the acidic variable region of isoform A and collagen type I at physiological temperature may contribute to the regulatory role of collagen type XI by stabilizing the interaction and facilitating the steric hindrance by the Npp domain of isoform A to regulate further lateral aggregation of collagen type I molecules on the growing fibrils, especially during the growth phase.

**Figure 6:**
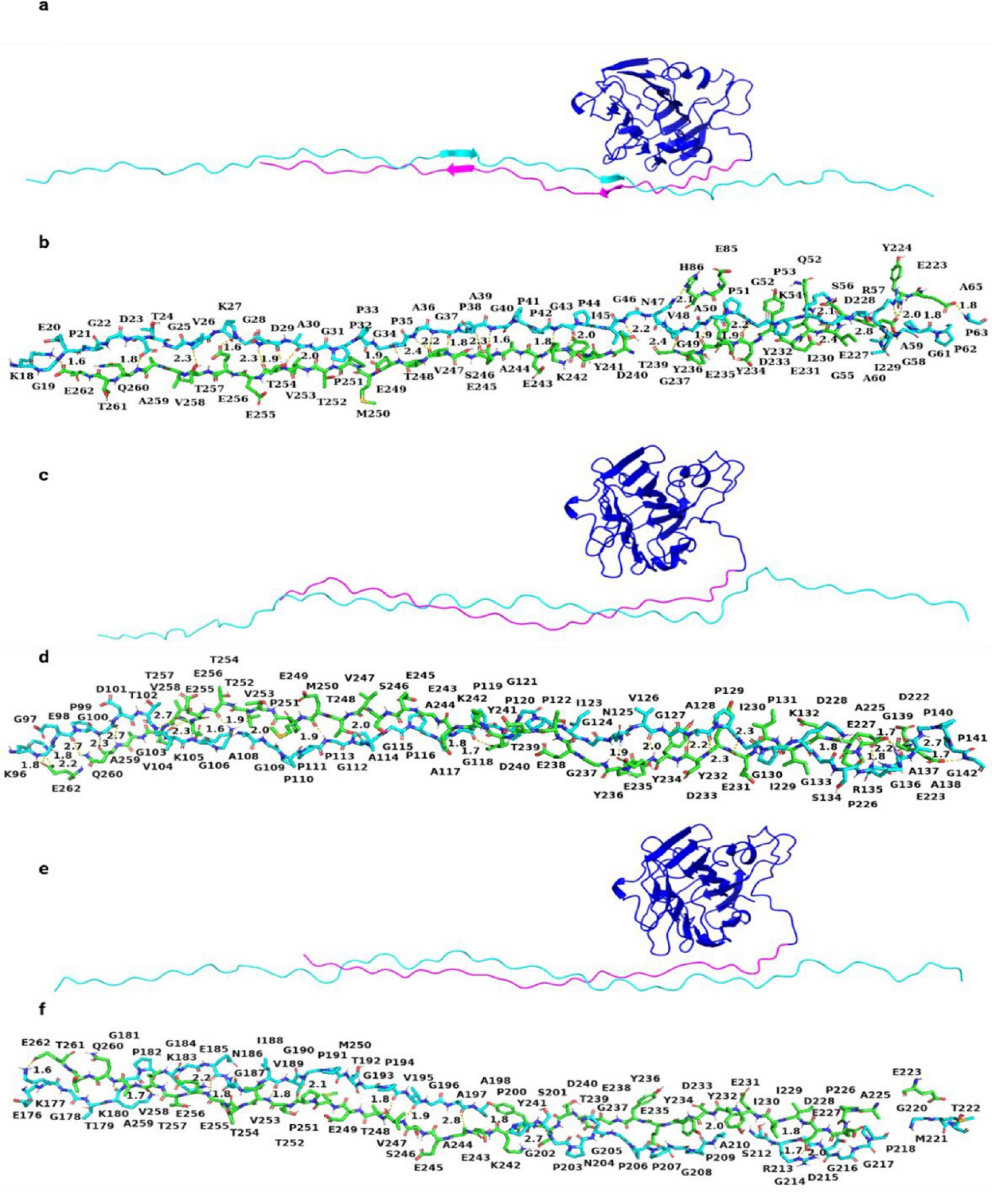
Interaction between collagen α1(XI) NTD isoform A and collagen type I. (a), (c), and (e) represent the alignment between three chains of collagen type I and isoform A (Npp (blue), variable region (pink), and collagen type I (cyan)). (b), (d), and (f) represent interacting amino acid residues (green represents isoform A and cyan represents collagen type I). (d) aa 79–156 of the α1 chain and (f) aa 157–234 of the α2 chain.

**Figure 7:**
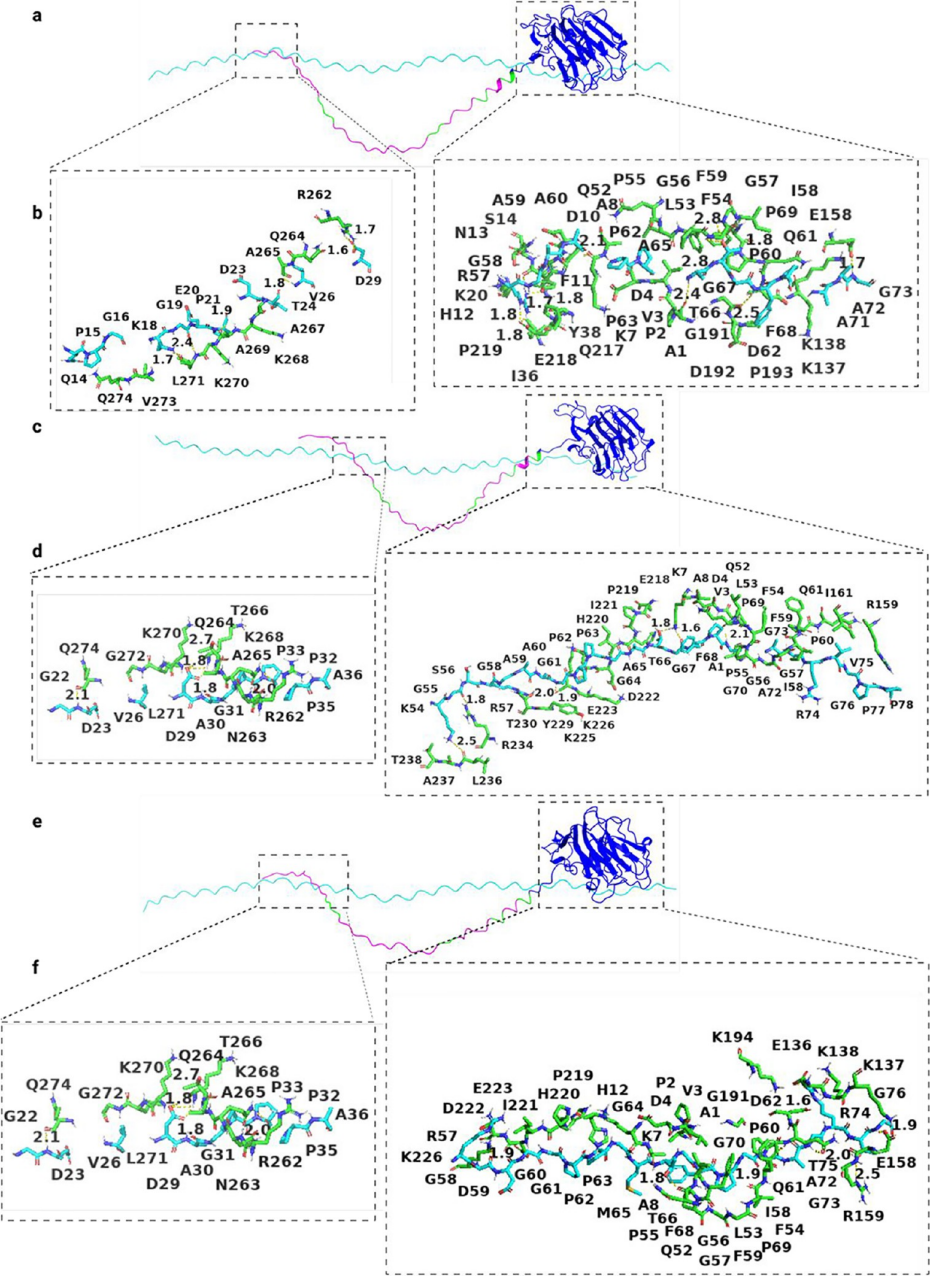
Interaction between collagen α1(XI) NTD isoform B and collagen type I. (a), (c), and (e) represent the alignment between three chains of collagen type I and isoform B (Npp (blue), variable region (pink), triple lysine motifs (lime), and collagen type I (cyan)). (b), (d), and (f) represent interacting amino acid residues (green represents isoform A and cyan represents collagen type I).

**Figure 8:**
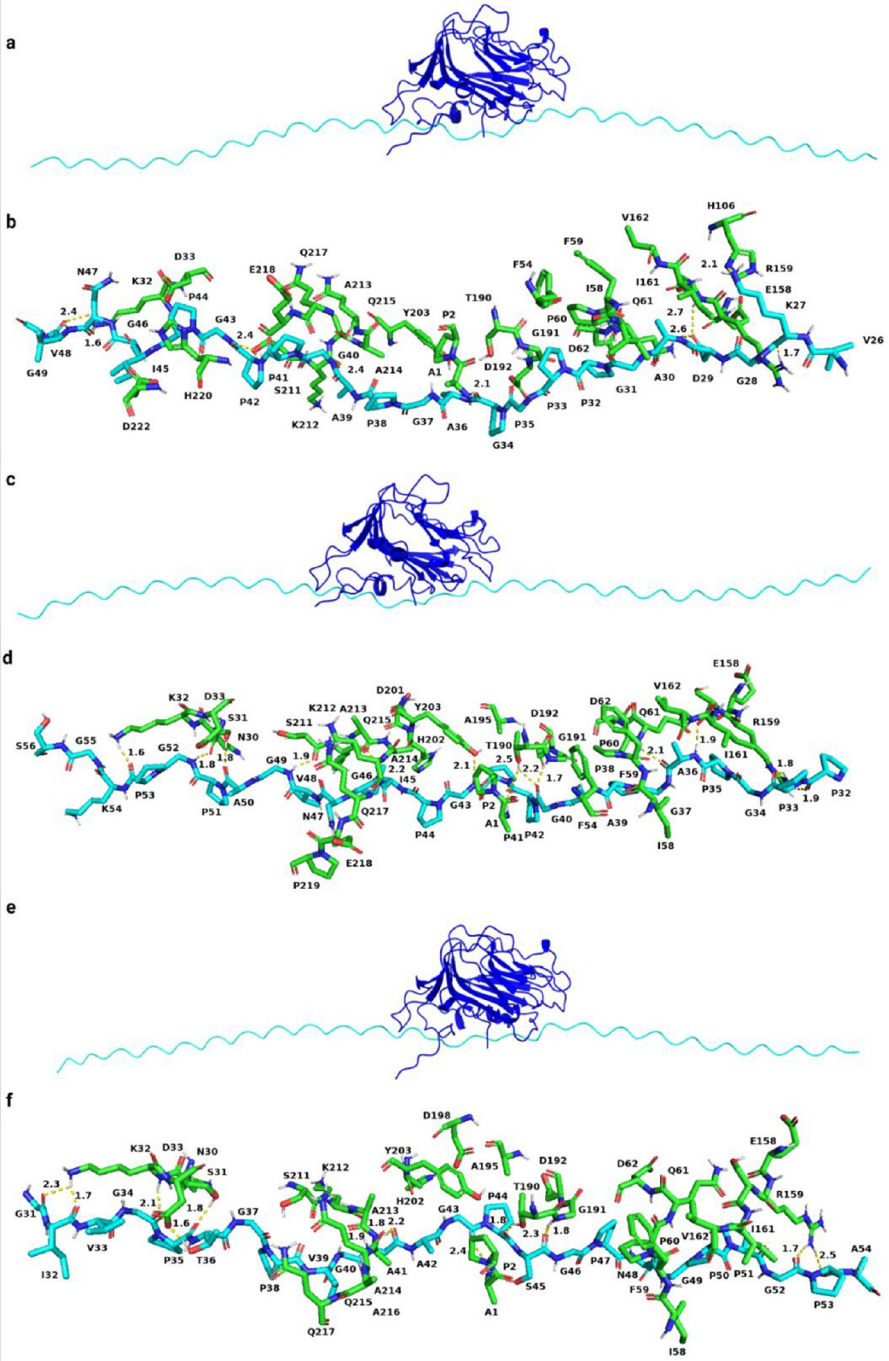
Interaction between collagen α1(XI) NTD isoform 0 and collagen type I. (a), (c), and (e) represent the alignment between three chains of collagen type I and isoform 0 (Npp (blue), variable region (pink), and collagen type I (cyan)). (b), (d), and (f) represent interacting amino acid residues (green represents isoform 0 and cyan represents collagen type I).

Figures 6–8 present the results of the protein–protein docking study using HADDOCK for each of the three isoforms. The results suggested that the acidic variable region of isoform A was closely aligned with the surface of collagen type I (Figure 6). However, for isoform B, the basic amino acid residues of the variable region maintained an unfavorable interaction with collagen type I (Figure 7). Isoform 0 binds to collagen type I through the amino acid interactions shown in Figure 8.

A previous study also suggested the linear alignment pattern of isoform A, whereas isoform B was readily accessible to its respective antibody in unmasked or undisrupted frozen tissue sections.^41^ For isoform A, the results from self-assembly kinetics suggested that the rate of fibrillogenesis decreased for both the lag phase and the growth phase compared to the control, whereas the activation energy for both the lag phase and growth phase increased (Figures 2 and 3). In the case of isoform 0, during the lag phase, the rate was higher compared to that of the control, accompanied by a lower activation energy. In contrast, during the growth phase of isoform 0, the rate was low, and the activation energy was high (Figures 2 and 3). The rate constant for isoform B increased significantly at 30 and 35 °C similar to isoform 0 during the lag phase from the rate constant at 25 °C, whereas in the growth phase, the rate increased steadily (Figure 2 and Supplemental Tables 3 and 4). The activation energy for isoform B during the lag phase was higher than both the control and isoform 0 but lower than isoform A. Additionally, during the growth phase, the activation energy for isoform B was higher than for the control but lower than both isoforms A and 0. This phenomenon may be attributed to unique interactions that occur between isoform B and collagen type I. In contrast, the growth phase followed a linear relationship between the rate of fibrillogenesis and activation energy as per the Arrhenius equation. The reason behind this phenomenon in the lag phase could be due to the formation of amorphous complexes between isoform B and collagen type I at lower temperatures.^42^ For isoform B, in terms of bonding dynamics, we noted a higher frequency of hydrogen bonds and salt-bridge formation at 25 °C compared to 37 °C during the 100 ns time course (Figures 4c,d and 5c,d).

The results from the MD simulation suggest that a more favorable interaction takes place between the acidic variable region of isoform A and collagen type I compared to isoforms B and 0. In addition to the interaction with the variable region, the amino acid residues from the Npp domain also interact with collagen type I. As a result of these interactions, the Npp domain may bind to the surface of collagen type I with a relatively high affinity, as depicted in Figure 6. Therefore, the Npp may impose steric hindrance to the further deposition of collagen molecules. Steric hindrance may, in turn, reduce the rate of self-assembly of collagen type I and increase the activation energy for both the lag phase and growth phase when compared to controls (Figures 2 and 3).

**Figure 9:**
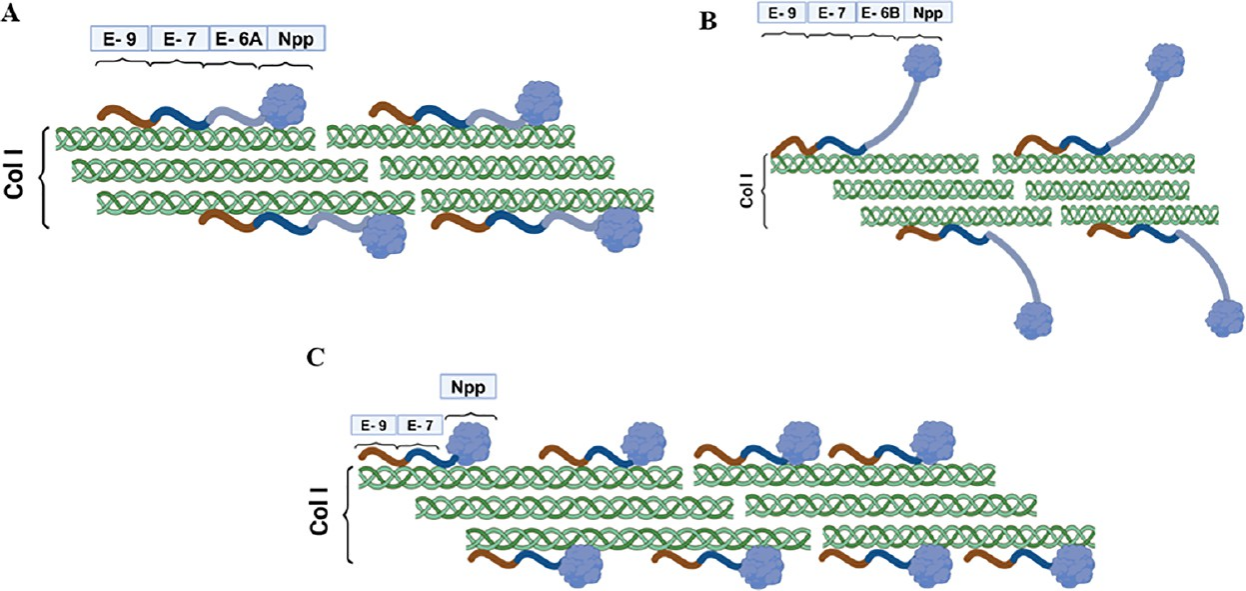
Interactions of collagen type I and collagen α1(XI) NTD isoforms: (A) isoform A and collagen type I interaction, (B) isoform B and collagen type I interaction, and (C) isoform 0 and collagen type I interaction.

Building upon the experimental findings of our study, a model of collagen type I and collagen α1(XI) isoform binding is presented in Figure 9.

Isoform B possesses exon 6B, which is highly basic. From the MD simulation and protein–protein docking results, it is evident that this basic segment of the protein demonstrated an unfavorable interaction with collagen type I. This property of isoform B may keep the Npp domain away from the surface of collagen type I under physiologic ionic conditions and temperature or potentially destabilize the interaction. As a result, the Npp domain of isoform B may create relatively less steric hindrance for further deposition of collagen type I molecules to the surface of growing fibrils, as shown in Figure 9B. Isoform 0 participates in a number of salt bridges (Figure 4e,f) and hydrogen bonds (Figure 5e,f), as shown by the MD simulation studies. Moreover, the docking studies also show interactions between isoform 0 and collagen type I (Figure 8).

The interaction of isoform 0 with collagen type I contributed to a slower rate for the growth phase and a higher activation energy compared to the control. This effect may be attributed to substantial steric hindrance by the Npp domain at the surface of the fibril, limiting further deposition of collagen type I molecules on the growing fibril. The highest activation energy for isoform 0 observed among the three isoforms could be attributed to the utilization of a high ratio of this recombinant protein to collagen type I, given that this isoform lacks any acidic or basic segment in the variable region. The observation of the slowest rate constant during the growth phase and the highest activation energy for isoform 0 may suggest inhibition of growth by a mechanism based on steric hindrance. Drawing inspiration from the experimental results for isoform 0, we put forth a model detailing the arrangement between isoform 0 and collagen type I in Figure 9C. The outcomes of the in vitro self-assembly kinetics of the three isoforms align with the initial hypothesis, suggesting that the amino-terminal domain cannot be accommodated within the fibril. Thus, the resulting steric hindrance from this substantial globular domain is thought to prevent further inclusion of collagen fibrils. Due to the differential chemical nature of the variable regions of different isoforms, the rate of fibril assembly and thermodynamic parameters may also change.

The binding energy calculation of the protein–protein interaction is an important measure to predict the mode of interaction. High electrostatic interactions were observed for isoforms A and 0. However, for isoform B, this is quite the opposite (Supplemental Figure 3 and Table 5). However, the van der Waals interaction also contributed to the affinity which was highest for isoform 0 and lowest for isoform B. The bonded interactions were very negligible for all three isoforms (Supplemental Table 5). The contact maps show that isoform A has a higher contact frequency (Supplemental Figures 4 and 5) compared to isoform B (Supplemental Figures 6 and 7) within the variable region of the respective isoforms. Furthermore, the contact map for isoform A reveals that, compared to isoform B, there are more interactions between the Npp region (amino acids 1–223, Supplemental Figures 4 and 5 for isoform A and Supplemental Figures 6 and 7 for isoform B) with the three chains of collagen type I. This phenomenon may be due to the favorable interactions of isoform A, which may increase the persistence of the NTD on the surface of the growing fibril. Supplemental Figures 8 and 9 depict isoform 0 interactions with the triple helical chain of collagen type 1. Contact maps show that interactions are different for each of the isoforms and indicate that the variable region plays an important role in the mechanism of interaction with collagen type I.

The interactions between the isoforms and collagen type I take place due to hydrogen bonds, salt bridges, and van der Waals interaction. In addition, we observed the highest COM distance for isoform B interacting with collagen type I, indicating the lowest extent of protein–protein interaction at 37 °C, followed by isoform A and then isoform 0. Similarly, for the SASA, we observed the lowest SASA for isoform 0 interacting with collagen type I, indicative of the strongest protein–protein interaction among the three isoforms, followed by isoform A and then isoform B (Supplemental Figure 2).

**Figure 10:**
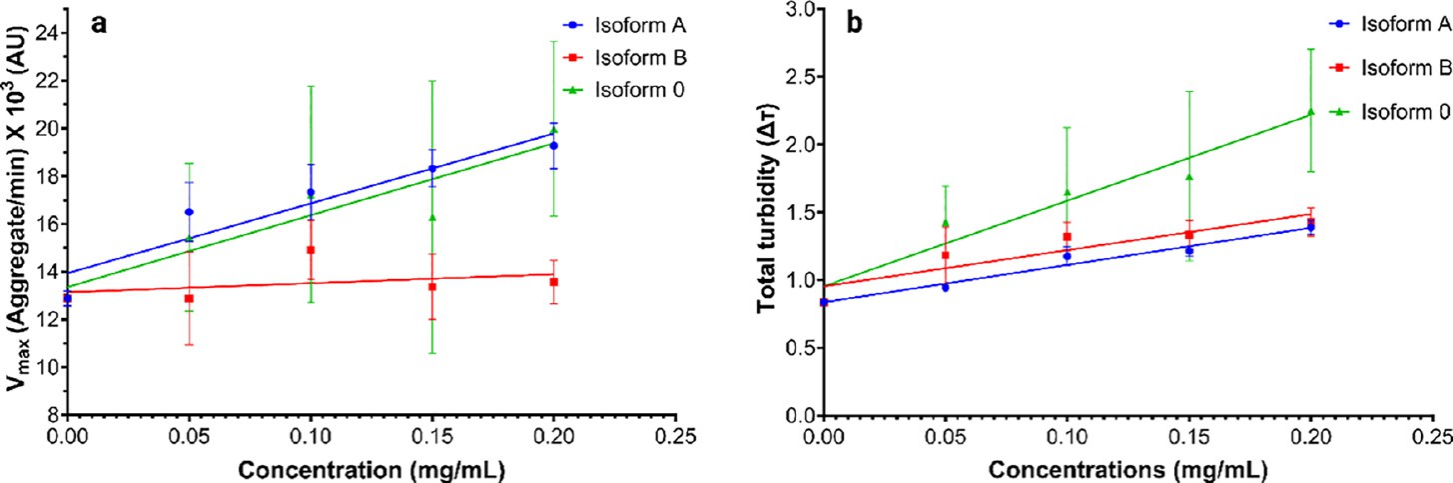
Effect of collagen α1(XI) NTD isoforms on the fibril formation of collagen type I. (a) Velocity max as a function of isoform concentration at 25 °C and 310 nm. (b) Total turbidity measurements were for isoforms A (blue), B (red), and 0 (green). Data points at 0 mg/mL represent control values (collagen type I only without NTD isoforms).

The aggregate/min versus concentration plot at 25 °C showed the transient rate of the growth phase increase up until 0.2 mg/mL for isoform A and isoform 0 (Figure 10a). Above this concentration, the aggregate/min versus concentration decreased. However, for isoform B, the rate did not increase significantly with increasing concentration. A similar trend was observed for total turbidity (Δτ) (Figure 10b). Thus, while the isoforms of NTD decreased the rate during the growth phase, they may increase the duration of the growth phase and the final total turbidity compared to controls.

Collagen type XI is essential for healthy skeletal development, and the mechanism may be distinct from the regulation of fibrillogenesis. For example, the de novo expression pattern of isoform B appears to be restricted to the periphery of the cartilage underlying the perichondrium of the diaphysis in the developing long bones. Isoform B has been shown to interact with BSP, which is the most potent nucleator of hydroxyapatite in bone, and interaction occurs near the gap region of collagen types I and II.^43,44^

**Figure 11:**
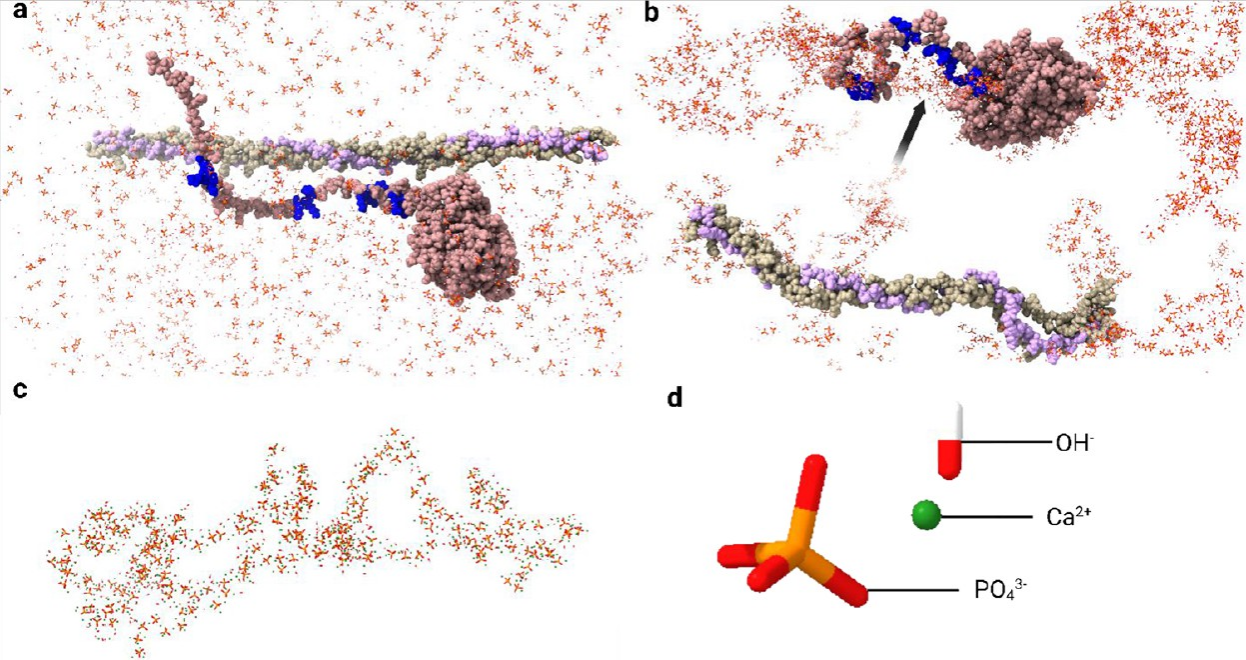
Ion cluster formation on the surface of isoform B is influenced by the basic amino acid residues. (a) Presence of PO_4_^3–^, OH^–^, and Ca^2+^ ions in water with collagen type I and collagen α1(XI) NTD isoform B at 0 ns simulation, with 4 triple lysine motifs shown in blue. (b) Ion cluster formation on the surface of isoform B at 100 ns influenced by the 4 triple lysine motifs (blue) and indicated with an arrow. (c) Ion clusters in the region of the four triple lysine motifs. (d) Identity of the constituent ions of hydroxyapatite is included in the MD simulation.

The nucleation of Ca–P mineralization takes place in the gap/hole region of collagen type I.^20,45^ An MD simulation with constitutive ions (PO_4_^3–^, OH^–^, and Ca^2+^) of hydroxyapatite (Ca_10_(PO_4_)_6_(OH)_2_) with collagen type I and collagen α1(XI) isoform B reveals that most of the accumulation of ions occurs around the charged amino acid residues, in addition to their presence in the solution (Figure 11). Isoform B contains four triple lysine motifs, resulting in the basic nature of this isoform. A distinct clustering was observed for the four triple lysine motifs as well as for the ion clusters that formed on the surface of isoform B by using MD (Figure 11b).

**Figure 12:**
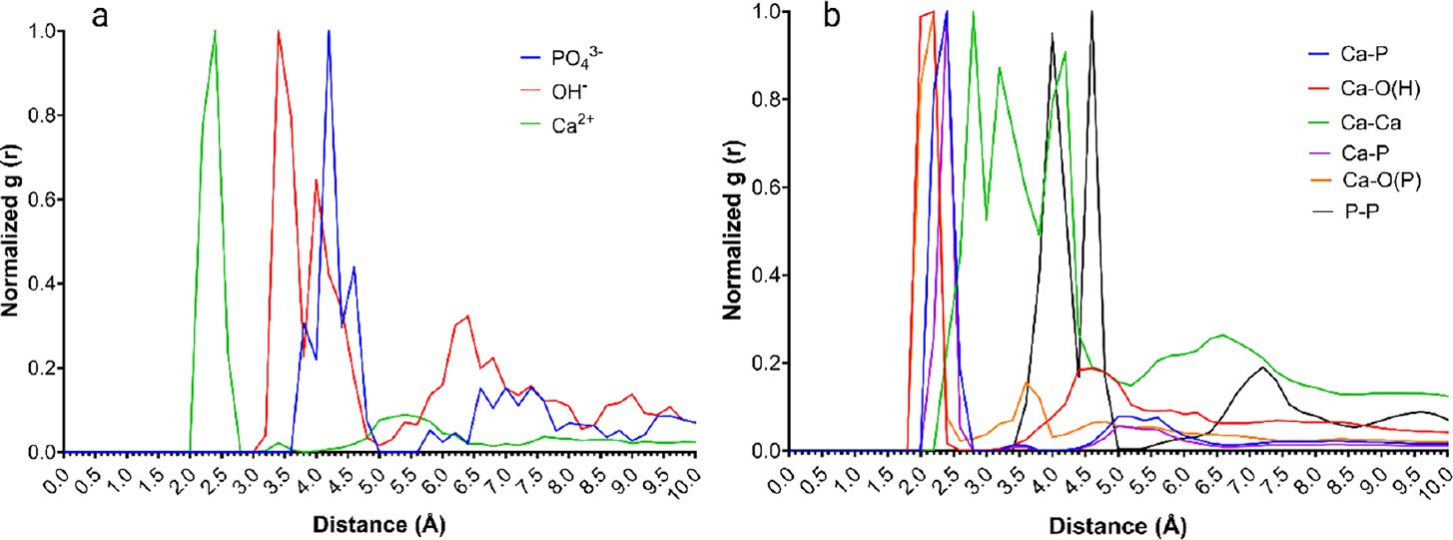
Radial distribution function of (a) distance of basic amino acid residues to deposited ions on the surface of collagen α1(XI) NTD isoform B. (b) Interparticle distances of the ion cluster on the surface of basic amino acid residues of isoform B.

The Npp may also participate in the formation of ion clusters; however, the proteolytic removal of the Npp domain from isoform B is more rapid compared to other isoforms.^13^ The radial distribution function shows close coordination among the basic amino acid residues and the three constitutive ions of hydroxyapatite (PO_4_^3–^, OH^–^, and Ca^2+^) (Figure 12a). Moreover, the interparticle distances (Figure 12b) of the accumulated ion clusters are consistent with previously published crystallographic data and MD simulation data.^46,47^

## Conclusions

- Collagen α1(XI) NTD isoforms modulate the collagen self-assembly process through molecular interactions that alter the rates of nucleation and growth. Isoform-specific differences result from unique bonding dynamics, potentially imparting varying degrees of steric hindrance due to the chemically distinct variable regions. A reduction in rate constants during the lag and/or growth phase of self-assembly may facilitate higher yields observed in the plateau phase.
- Isoform A interacts favorably with collagen type I due to interactions mediated by the acidic variable region. This interaction raises the activation energy of fibril growth while decreasing the rate constant. In contrast, the extremely basic variable region of isoform B results in an unfavorable interaction with collagen type I. This adverse interaction may contribute to the rapid proteolytic removal of the Npp domain that has been observed previously. Consequently, compared to isoform A, isoform B may result in less steric hindrance. This is supported by our findings of a lower activation energy for isoform B compared to isoform A. Lastly, during the growth phase, isoform 0 had the largest activation energy and the lowest rate constant, suggesting that the variable region controls Npp–collagen type I interaction and that the Npp domain alone is more efficient at inhibiting lateral growth of the collagen fibril than isoform A or B.
- An isoform-specific mechanism of regulating fibrillogenesis may also imply a tissue-specific modulation of collagen fibrillogenesis within the local microenvironments in both developing and mature tissues due to unique temporal and spatial expression patterns for each isoform. Such modulation may play a crucial role in influencing developmental processes and maintaining homeostasis throughout an organism’s lifespan, as well as in the case of collagen-related diseases.
- Additional biological roles for specific isoforms of collagen α1(XI) may include facilitation of biomineralization.
- Future directions will investigate the role of specific isoforms in a tissue-specific manner and their influence on the self-assembly processes of collagen I and II. These studies may provide insights into different diseases and disorders. This information may enable the design of better suited regenerative medicine and/or treatment approaches for connective tissue diseases and repair of damage to cartilage and other tissues.

## Supporting Information Available

Alphafold 2-derived structures of collagen α1(XI) NTD isoforms, COM distances and total SASA, free energy of binding, contact maps, turbidity–time curves, apparent rate constants, summary of proteins used in this study, collagen type I α1 and α2 sequences, rate constants in the lag and growth phase, Gromacs molecular mechanics Poisson–Boltzmann surface area results, parameters for MS simulation, and parameters for Gromacs molecular mechanics Poisson–Boltzmann surface area (PDF)

## Accession Codes

UNIPROT P02454 Collagen alpha-1(I) chain. UNIPROT PO2466 Collagen alpha-2(I) chain. NCBI AH003429.3 Collagen alpha 1(XI) chain.

## Abbreviations

collagen α1(XI): collagen type 11 alpha 1 chain
NTD: N-terminal domain
Npp: amino propeptide domain
VR: variable region
BSP: bone sialoprotein
*E*_a_: activation energy
PBS: phosphate-buffered saline
BCA assay: bicinchoninic acid assay
PDB: Protein Data Bank
MD: molecular dynamics
cys: cysteine
pI: isoelectric point
*NVT*: number of particles (*N*), system volume (*V*), and temperature (*T*) are constant/conserved
*NPT*: number of particles (*N*), system pressure (*P*), and temperature (*T*) are constant/conserved
PME: particle mesh Ewald
gmxMMPBSA: Gromacs molecular mechanics Poisson–Boltzmann surface area
SASA: solvent accessible surface area
