# Supplementary material for: Collagen Alpha 1(XI) Amino-Terminal Domain Modulates Type I Collagen Fibril Assembly
Source: Biochemistry. 2025 Jan 22;64(3):735–47. doi: 10.1021/acs.biochem.4c00434 (PMC11800387; doi:10.1021/acs.biochem.4c00434)
Supplement: Supplementary file 1 — bi4c00434_si_001.pdf [file bi4c00434_si_001.pdf]

# Supporting Information

Collagen alpha 1(XI) amino-terminal domain modulates type I collagen fibril assembly

*Abu Sayeed Chowdhury*[a] *and Julia Thom Oxford*[b]*

[a]Biomolecular Sciences Graduate Program, Boise State University, 1910 University Drive, Boise, Idaho, 83725 USA

[b]Biomolecular Research Institute, Boise State University, 1910 University Drive, Boise, Idaho, 83725-1511 USA

*Email:joxford@boisestate.edu

Alphafold 2 derived structures of collagen α1 (XI) NTD isoforms, Center of mass distances and total solvent accessible surface area, Free energy of binding, Contact maps, Turbidity-time curves, Apparent rate constants, Summary of proteins used in this study, Collagen type I α1 and α2 sequences, Rate constants at the lag and growth phase, Gromacs molecular mechanics Poisson-Boltzmann Surface Area results, Parameters for molecular dynamics simulation, Parameters for Gromacs molecular mechanics Poisson-Boltzmann Surface Area

**Table of contents: Figures**


| Contents | Page |
|---|---|
| Supplemental Figure 1: Alphafold 2 derived structures of collagen α1 (XI) NTD isoforms | S3 |
| Supplemental Figure 2: Center of mass distances and total solvent accessible surface areas | S4 |
| Supplemental Figure 3: Free energy of binding | S5 |
| Supplemental Figure 4: Contact map at 298 K for isoform A and collagen type I. | S6 |
| Supplemental Figure 5: Contact map at 310 K for isoform A and collagen type I | S7 |
| Supplemental Figure 6: Contact map at 298 K for isoform B and collagen type I | S8 |
| Supplemental Figure 7: Contact map at 310 K for isoform B and collagen type I | S9 |
| Supplemental Figure 8: Contact map at 298 K for isoform 0 and collagen type I | S10 |
| Supplemental Figure 9: Contact map at 310 K for isoform 0 and collagen type I | S11 |
| Supplemental Figure 10: Turbidity-time curve at 293 K +/- isoforms at 0.05 mg/mL | S12 |
| Supplemental Figure 11: Turbidity-time curve at 293 K +/- isoforms at 0.1 mg/mL | S12 |
| Supplemental Figure 12: Turbidity-time curve at 293 K +/- isoforms at 0.15 mg/mL | S13 |
| Supplemental Figure 13: Turbidity-time curve at 293 K +/- isoforms at 0.2 mg/mL | S13 |
| Supplemental Figure 14: Turbidity-time curve at 298 K +/- isoforms at 0.05 mg/mL | S14 |
| Supplemental Figure 15: Turbidity-time curve at 298 K +/- isoforms at 0.1 mg/mL | S14 |
| Supplemental Figure 16: Turbidity-time curve at 298 K +/- isoforms at 0.15 mg/mL | S15 |
| Supplemental Figure 17: Turbidity-time curve at 298 K +/- isoforms at 0.2 mg/mL | S15 |
| Supplemental Figure 18: Turbidity-time curve at 303 K +/- isoforms at 0.05 mg/mL | S16 |
| Supplemental Figure 19: Turbidity-time curve at 303 K +/- isoforms at 0.1 mg/mL | S16 |
| Supplemental Figure 20: Turbidity-time curve at 303 K +/- isoforms at 0.15 mg/mL | S17 |
| Supplemental Figure 21: Turbidity-time curve at 303 K +/- isoforms at 0.2 mg/mL | S17 |
| Supplemental Figure 22: Turbidity-time curve at 308 K +/- isoforms at 0.05 mg/mL | S18 |
| Supplemental Figure 23: Turbidity-time curve at 308 K +/- isoforms at 0.1 mg/mL | S18 |
| Supplemental Figure 24: Turbidity-time curve at 308 K +/- isoforms at 0.15 mg/mL | S19 |
| Supplemental Figure 25: Turbidity-time curve at 308 K +/- isoforms at 0.2 mg/mL | S19 |
| Supplemental Figure 26: Apparent rate constant for control at lag phase | S20 |
| Supplemental Figure 27: Apparent rate constant for control at growth phase | S20 |
| Supplemental Figure 28: Apparent rate constant for isoform A at lag phase | S21 |
| Supplemental Figure 29: Apparent rate constant for isoform A at growth phase | S21 |
| Supplemental Figure 30: Apparent rate constant for isoform B at lag phase | S22 |
| Supplemental Figure 31: Apparent rate constant for isoform B at growth phase | S22 |
| Supplemental Figure 32: Apparent rate constant for isoform 0 at lag phase | S23 |
| Supplemental Figure 33: Apparent rate constant for isoform 0 at growth phase | S23 |


**Supplemental Tables**


| Contents | Page |
|---|---|
| Supplemental Table 1: Summary of proteins used in this study | S24 |
| Supplemental Table 2: Collagen type I α1 and α2 sequences | S24 |
| Supplemental Table 3: Rate constants at the lag phase | S24 |
| Supplemental Table 4: Rate constants at the growth phase | S25 |
| Supplemental Table 5: Gromacs molecular mechanics Poisson-Boltzmann Surface Area results | S25 |
| Supplemental Table 6: Parameters for molecular dynamics simulation | S26 |
| Supplemental Table 7: Parameters for Gromacs molecular mechanics Poisson-Boltzmann Surface Area (gmx_mmpbsa) | S27 |

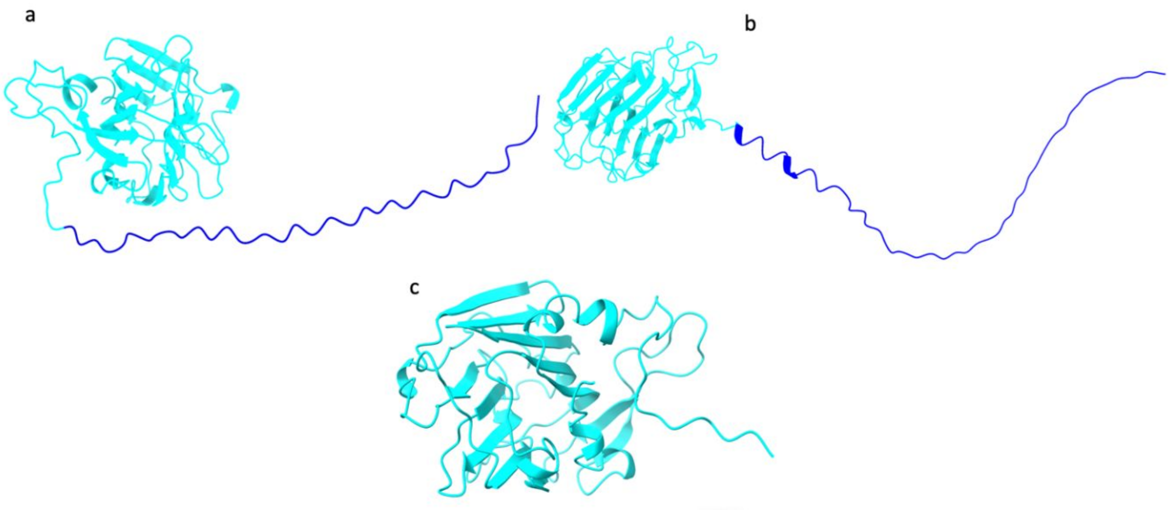


**Supplemental Figure 1:** Alphafold 2 derived structure of collagen α1 (XI) NTD isoforms. (a) Isoform A, (b) isoform B, and (c) isoform 0. The amino-propeptide domain of the NTD is colored cyan, and the variable regions of isoform A (a) and isoform B (b) are colored blue.

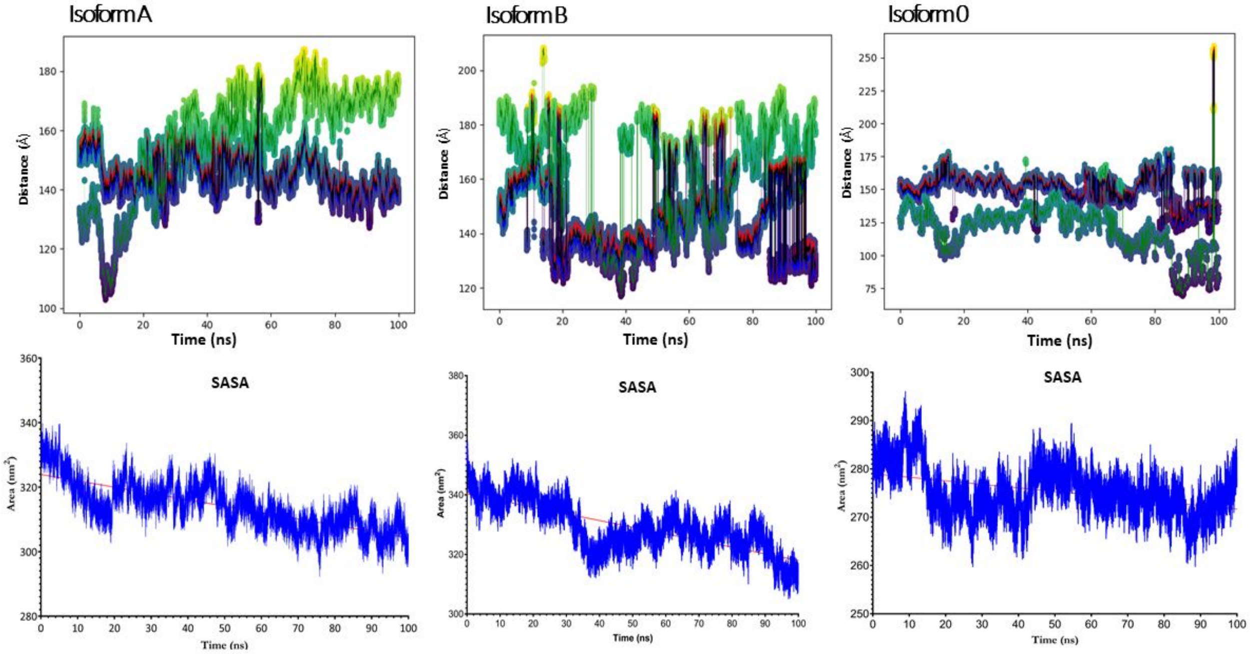


**Supplemental Figure 2**: Center of mass distances and total solvent accessible surface area. **Top panel:** Center of mass (COM) distances. COM of isoforms A, B, and 0 with collagen type I. Green indicates collagen $\alpha$1 (XI) NTD, red and blue indicates the two collagen I $\alpha$1 chains, and black indicates the $\alpha$2 chain. **Bottom panel:** The total solvent accessible surface area (SASA) of collagen $\alpha$1 (XI) NTD isoforms with collagen type I.

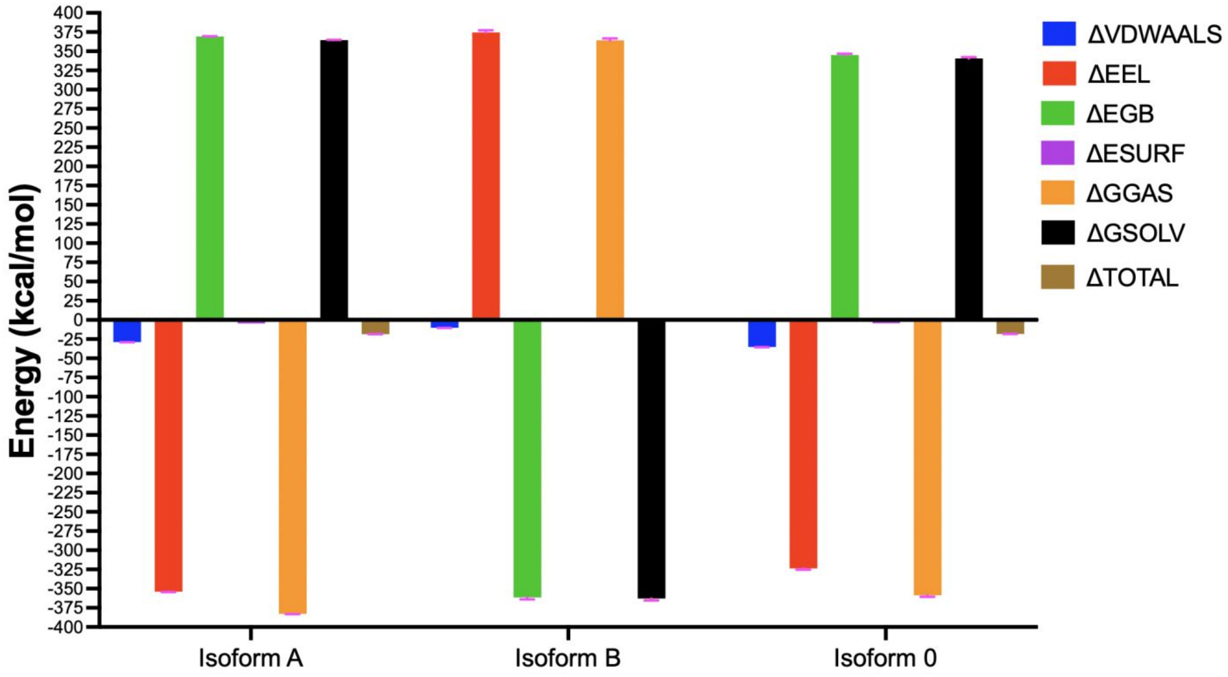


**Supplemental Figure 3:** Free energy of binding. Free energy of binding between collagen α1(XI) isoforms A, B, and 0 and collagen type I. (ΔVDWAALS- change in van der Waals energy, ΔEEL- change in electrostatic energy, ΔEGB- change in Generalized Born energy, ΔESURF- change in surface area energy, ΔGGAS- change in gas-phase energy, ΔGSOLV- change in solvation energy)

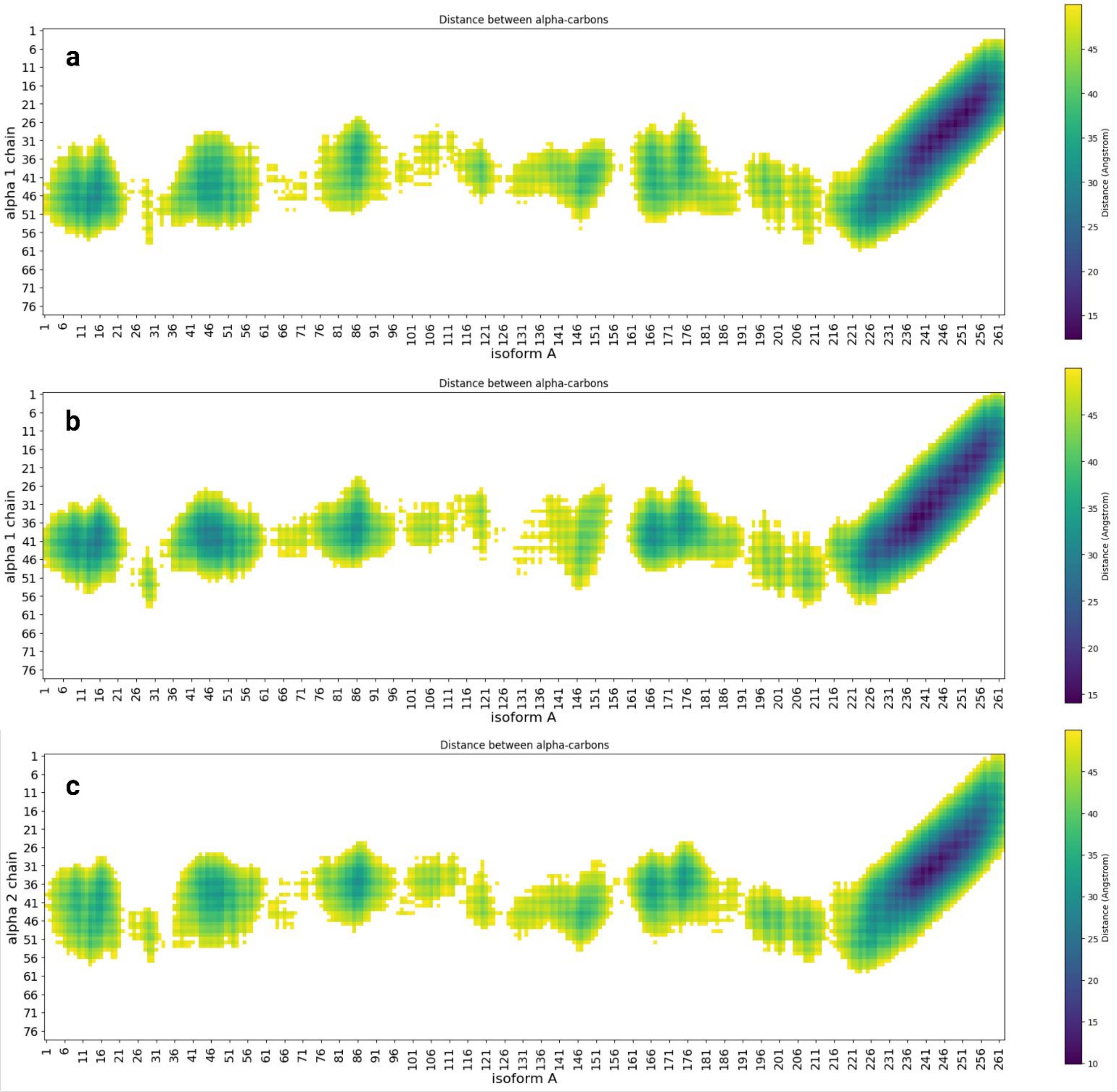


**Supplemental Figure 4:** Contact map at 298 K for isoform A and collagen type I. Contact distances between the three chains of collagen type I and isoform A with a cut-off value of 50 Å at 298 K, (a). & (b). Contact map between two alpha 1 chains and isoform A individually, (c). Contact map between alpha 2 chain of collagen type I and isoform A. Numbers on x-axis refer to amino acids of isoform A.

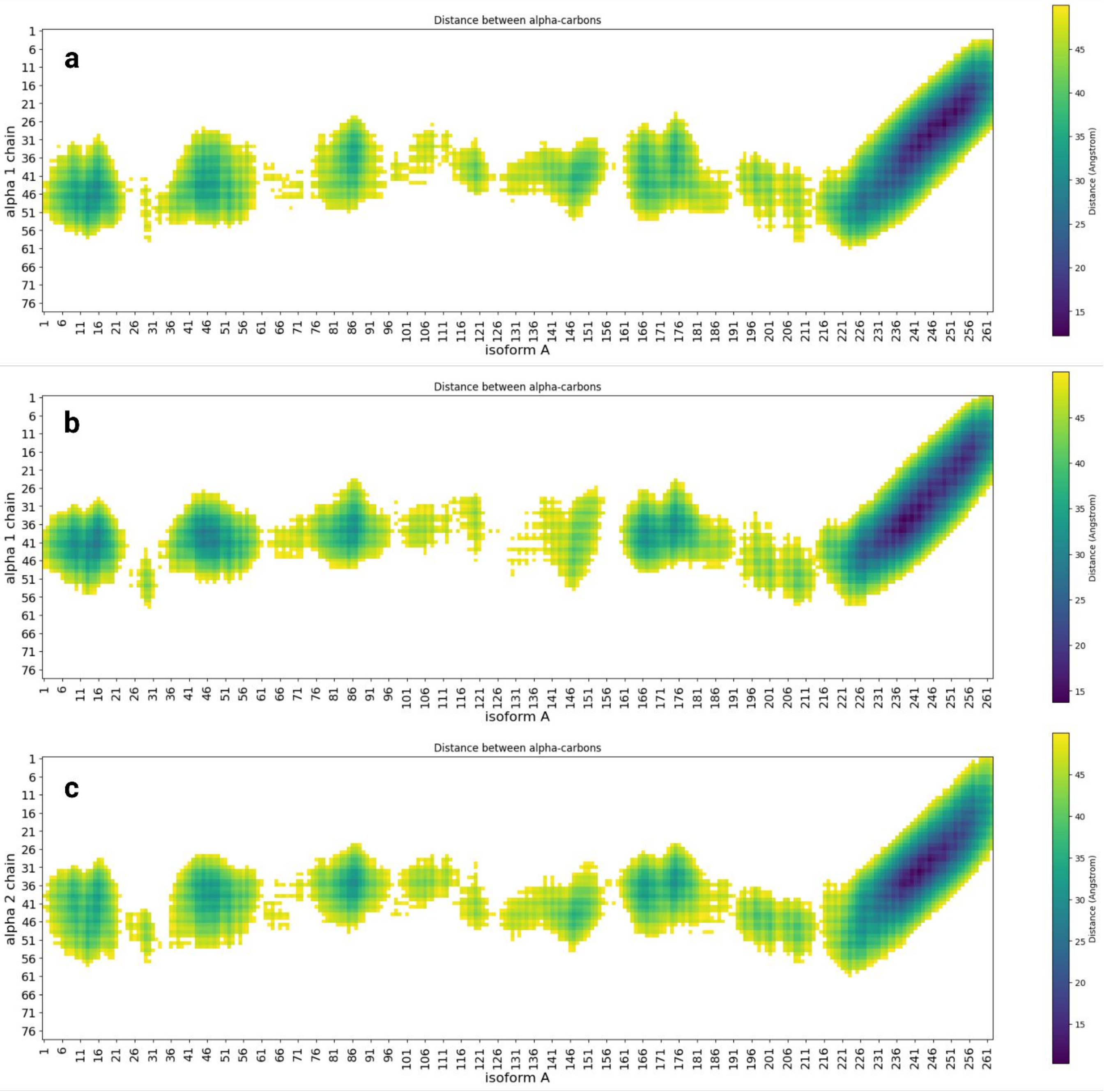


**Supplemental Figure 5:** Contact map at 310 K for isoform A and collagen type I. Contact map between the three chains of collagen type I and isoform A with a cut-off value of 50 Å at 310 K, (a). & (b). Contact map between two alpha 1 chains and isoform A individually, (c). Contact map between alpha 2 chain of collagen type I and isoform A. Numbers on x-axis refer to amino acids of isoform A.

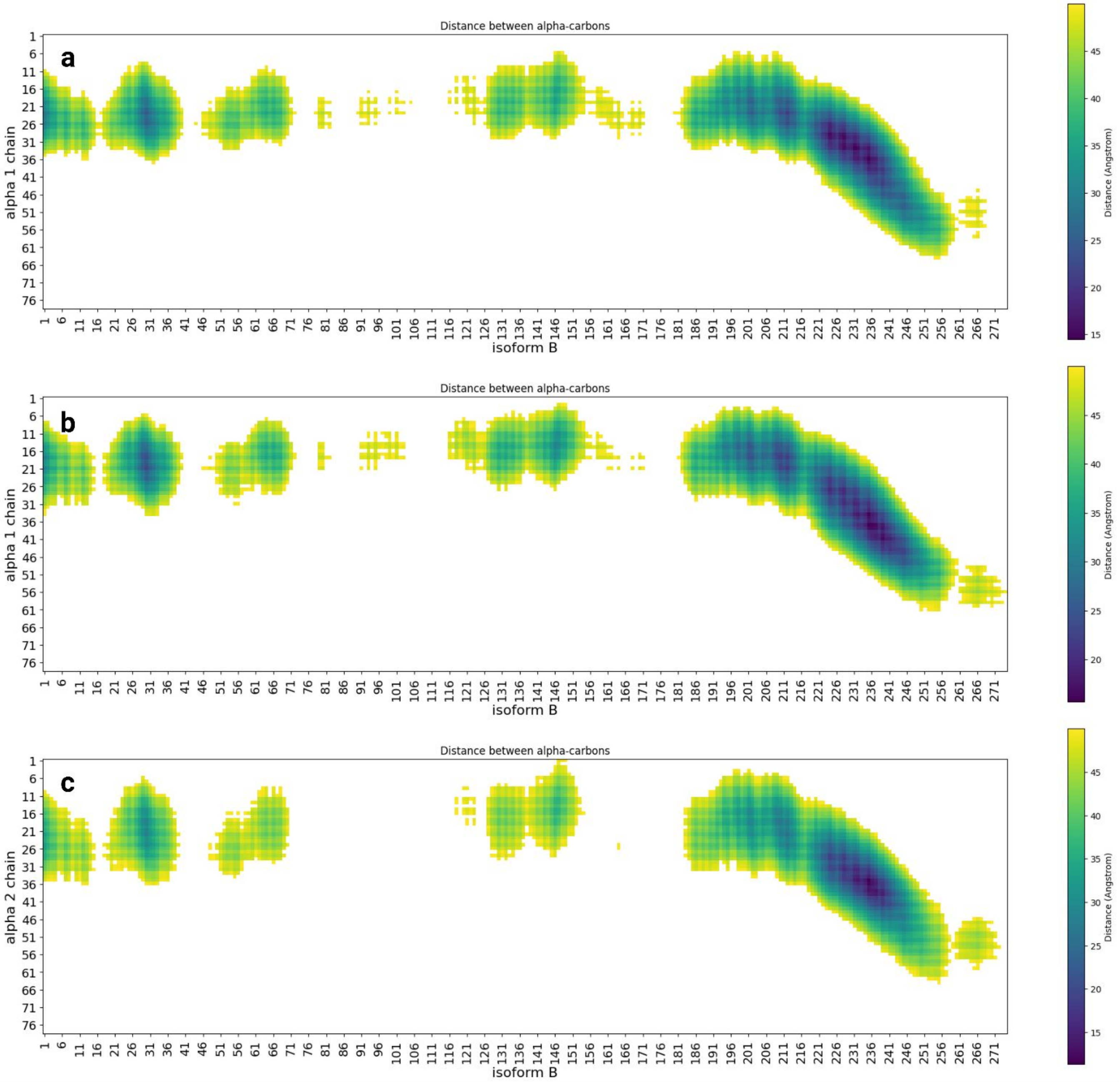


**Supplemental Figure 6:** Contact map at 298 K for isoform B and collagen type I. Contact distances between the three chains of collagen type I and isoform B with a cut-off value of 50 Å at 298 K, (a). & (b). Contact map between two alpha 1 chains and isoform B individually, (c). Contact map between alpha 2 chain of collagen type I and isoform B. Numbers on x-axis refer to amino acids of isoform B.

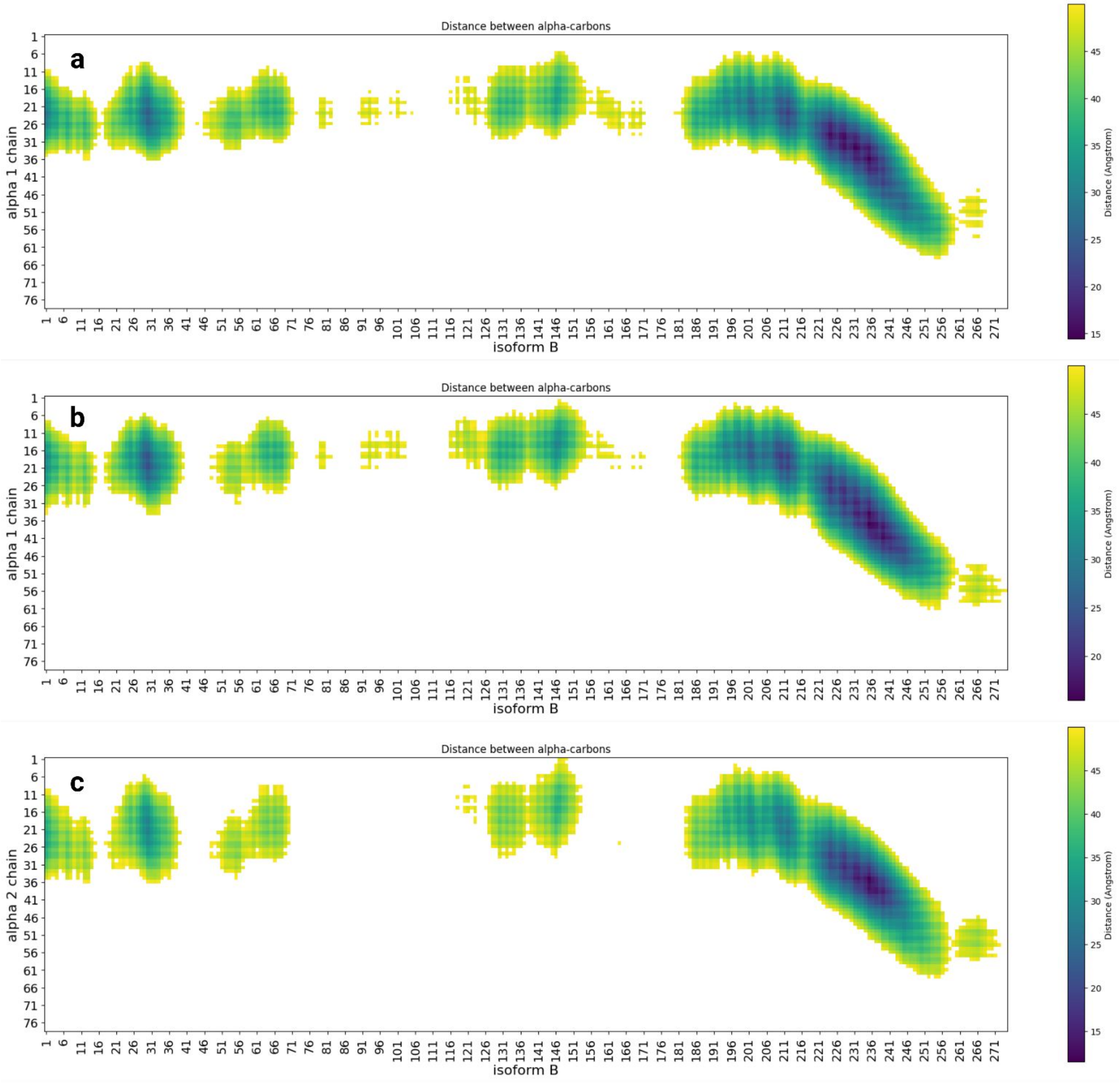


**Supplemental Figure 7:** Contact map at 310 K for isoform B and collagen type I. Contact distances between the three chains of collagen type I and isoform B with a cut-off value of 50 Å at 310 K, (a). & (b). Contact map between two alpha 1 chains and isoform B individually, (c). Contact map between alpha 2 chain of collagen type I and isoform B. Numbers on x-axis refer to amino acids of isoform B.

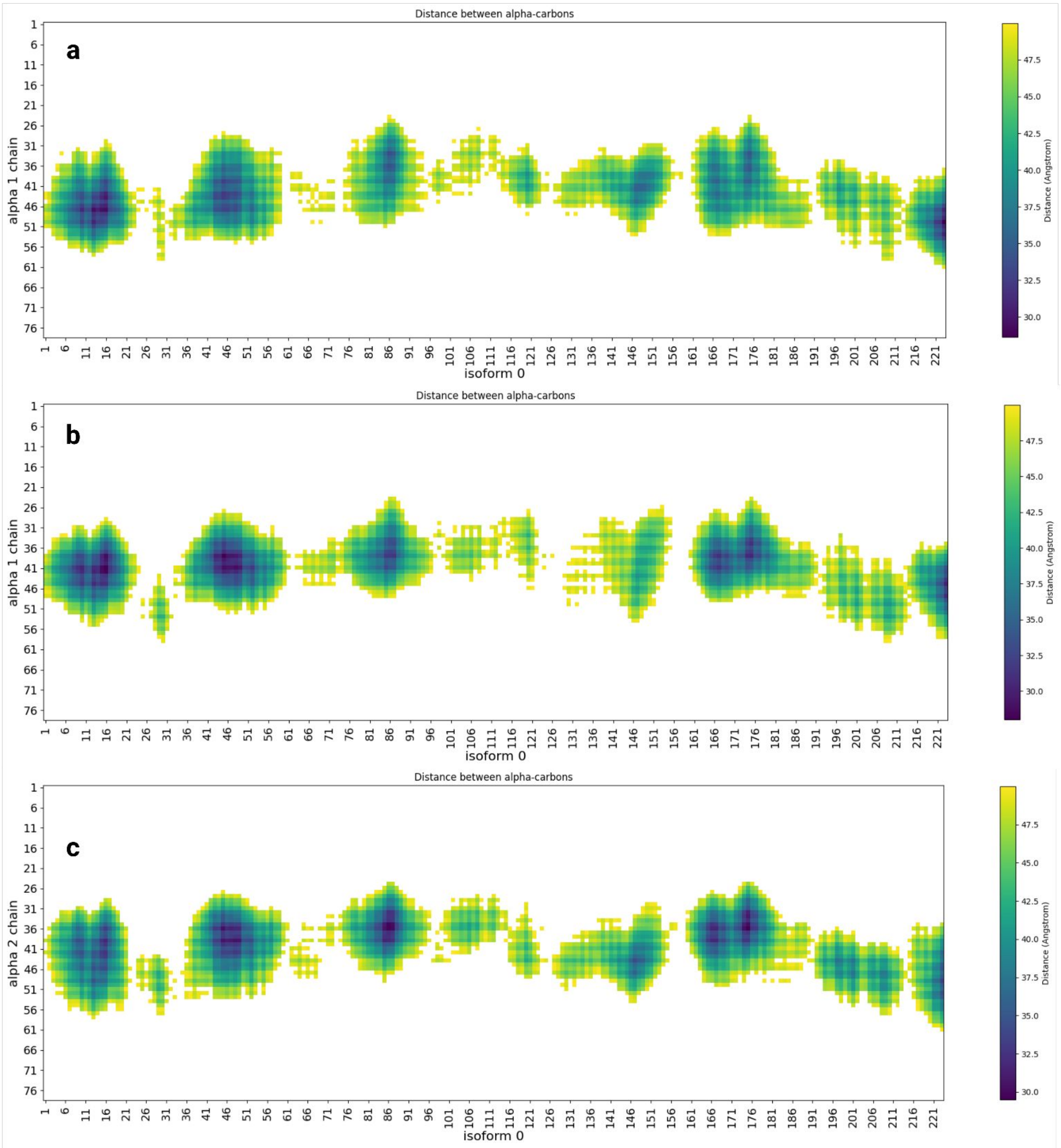


**Supplemental Figure 8:** Contact map at 298 K for isoform 0 and collagen type I. Contact distances between the three chains of collagen type I and isoform 0 with a cut-off value of 50 Å at 298 K, (a). & (b). Contact map between two alpha 1 chains and isoform 0 individually, (c). Contact map between alpha 2 chain of collagen type I and isoform 0. Numbers on x-axis refer to amino acids of isoform 0.

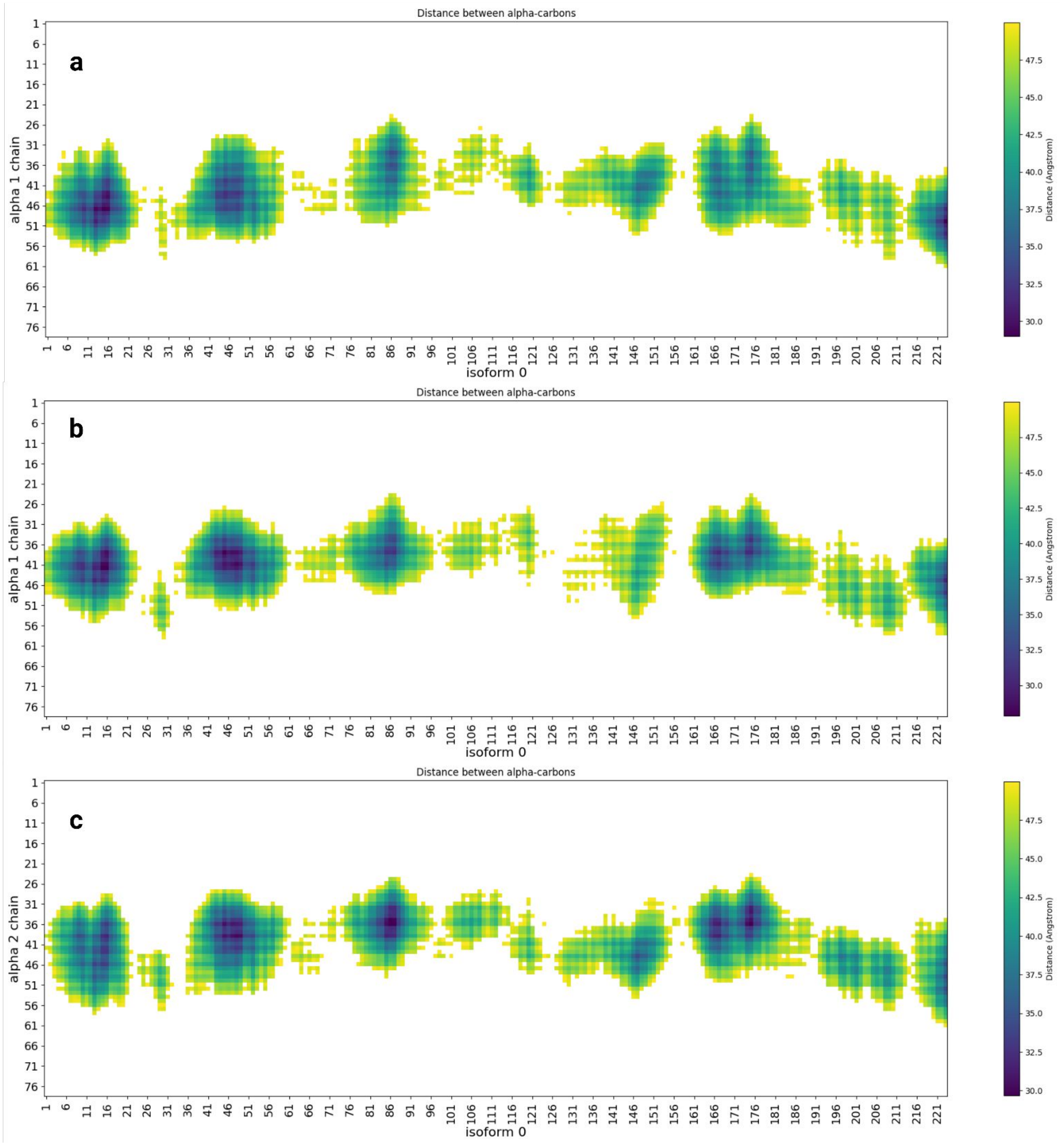


**Supplemental Figure 9:** Contact map at 310 K for isoform 0 and collagen type I. Contact distances between the three chains of collagen type I and isoform 0 with a cut-off value of 50 Å at 310 K, (a). & (b). Contact map between two alpha 1 chains and isoform 0 individually, (c). Contact map between alpha 2 chain of collagen type I and isoform 0. Numbers on x-axis refer to amino acids of isoform 0.

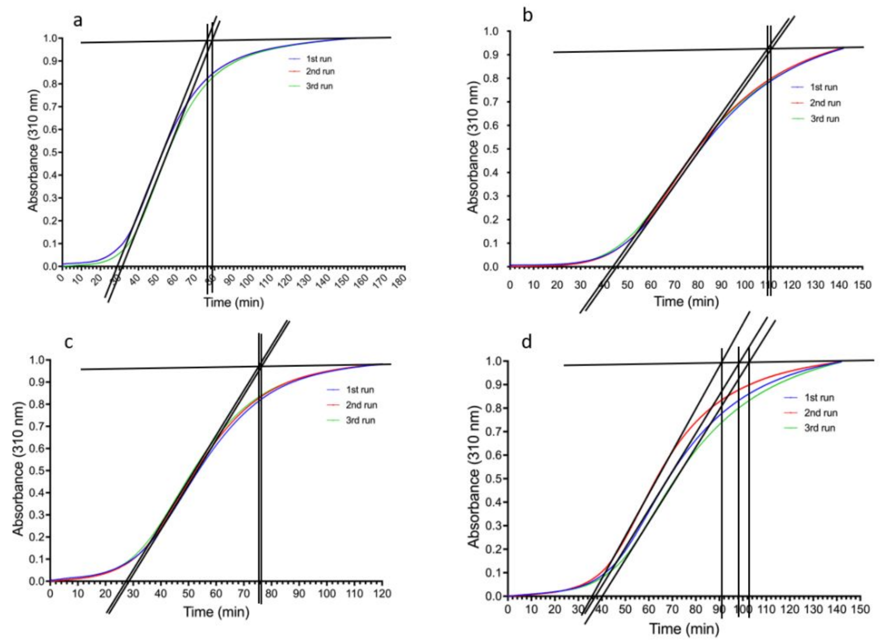


**Supplemental Figure 10:** Turbidity-time curve at 293 K +/- isoforms at 0.05 mg/mL. (a). 0.15 mg/mL collagen type I, (b). 0.15 mg/mL collagen type I with 0.05 mg/mL isoform A, (c). 0.15 mg/mL collagen type I with 0.05 mg/mL isoform B, and (d). 0.15 mg/mL collagen type I with 0.05 mg/mL isoform 0.

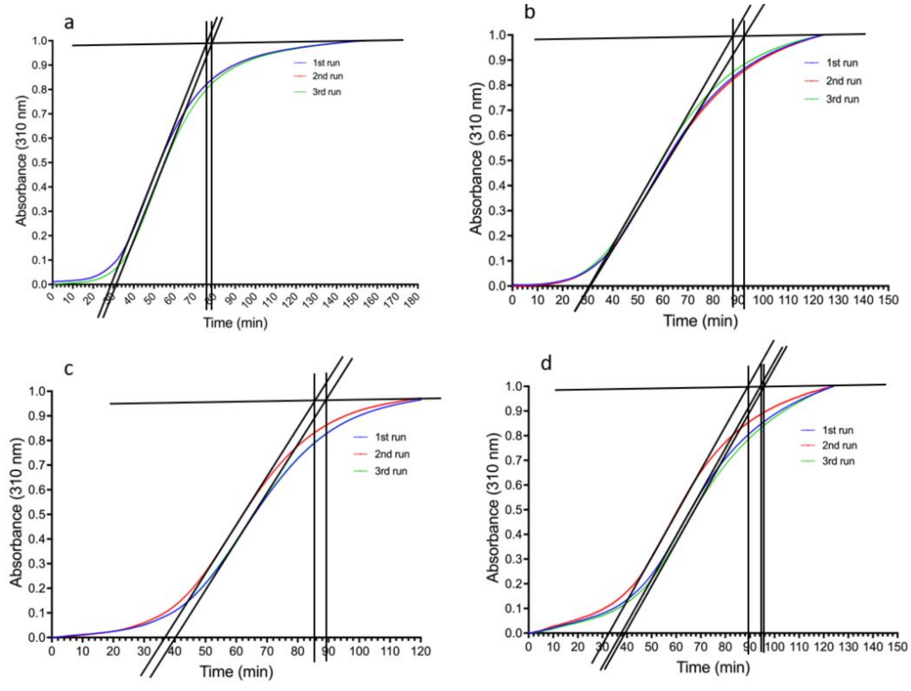


**Supplemental Figure 11:** Turbidity-time curve at 293 K +/- isoforms at 0.1 mg/mL. (a). 0.15 mg/mL collagen type I, (b). 0.15 mg/mL collagen type I with 0.1 mg/mL isoform A, (c). 0.15 mg/mL collagen type I with 0.1 mg/mL isoform B, and (d). 0.15 mg/mL collagen type I with 0.1 mg/mL isoform 0.

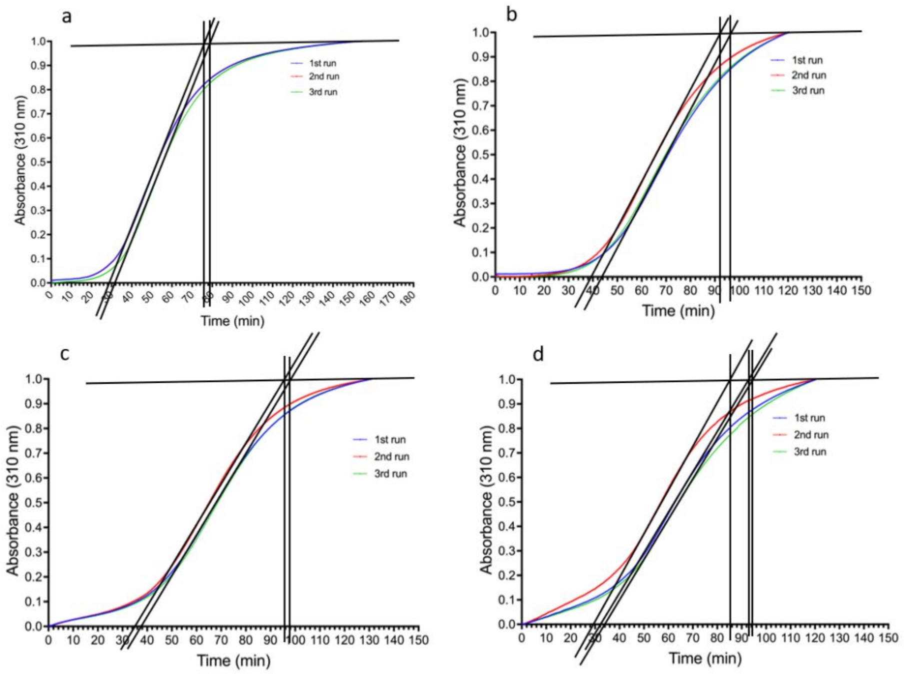


**Supplemental Figure 12:** Turbidity-time curve at 293 K +/- isoforms at 0.15 mg/mL. (a). 0.15 mg/mL collagen type I, (b). 0.15 mg/mL collagen type I with 0.15 mg/mL isoform A, (c). 0.15 mg/mL collagen type I with 0.15 mg/mL isoform B, and (d). 0.15 mg/mL collagen type I with 0.15 mg/mL isoform 0.

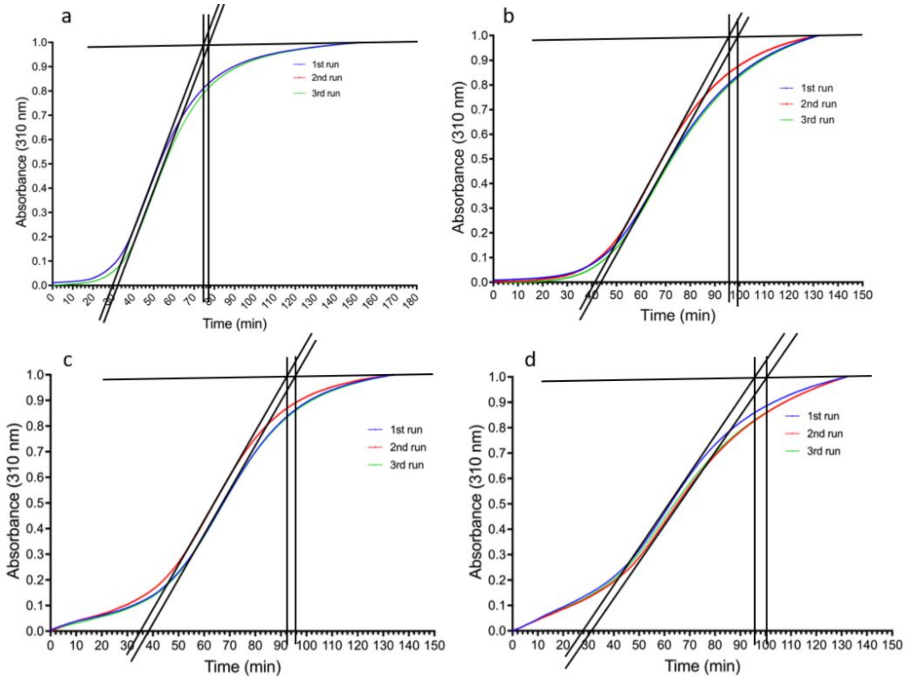


**Supplemental Figure 13:** Turbidity-time curve at 293 K +/- isoforms at 0.2 mg/mL. (a). 0.15 mg/mL collagen type I, (b). 0.15 mg/mL collagen type I with 0.2 mg/mL isoform A, (c). 0.15 mg/mL collagen type I with 0.2 mg/mL isoform B, and (d). 0.15 mg/mL collagen type I with 0.2 mg/mL isoform 0.

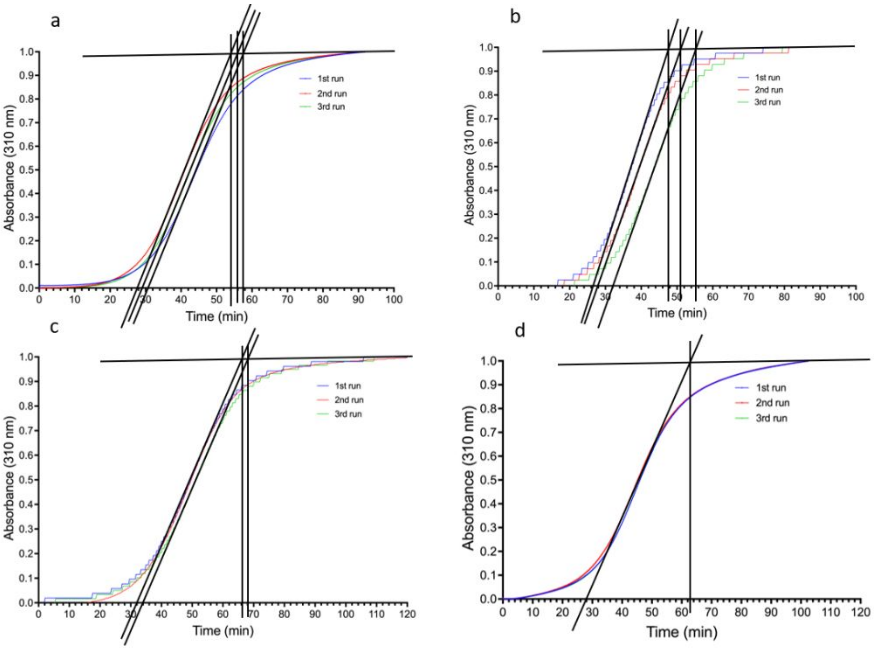


**Supplemental Figure 14:** Turbidity-time curve at 298 K +/- isoforms at 0.05 mg/mL. (a). 0.15 mg/mL collagen type I, (b). 0.15 mg/mL collagen type I with 0.05 mg/mL isoform A, (c). 0.15 mg/mL collagen type I with 0.05 mg/mL isoform B, and (d). 0.15 mg/mL collagen type I with 0.05 mg/mL isoform 0.

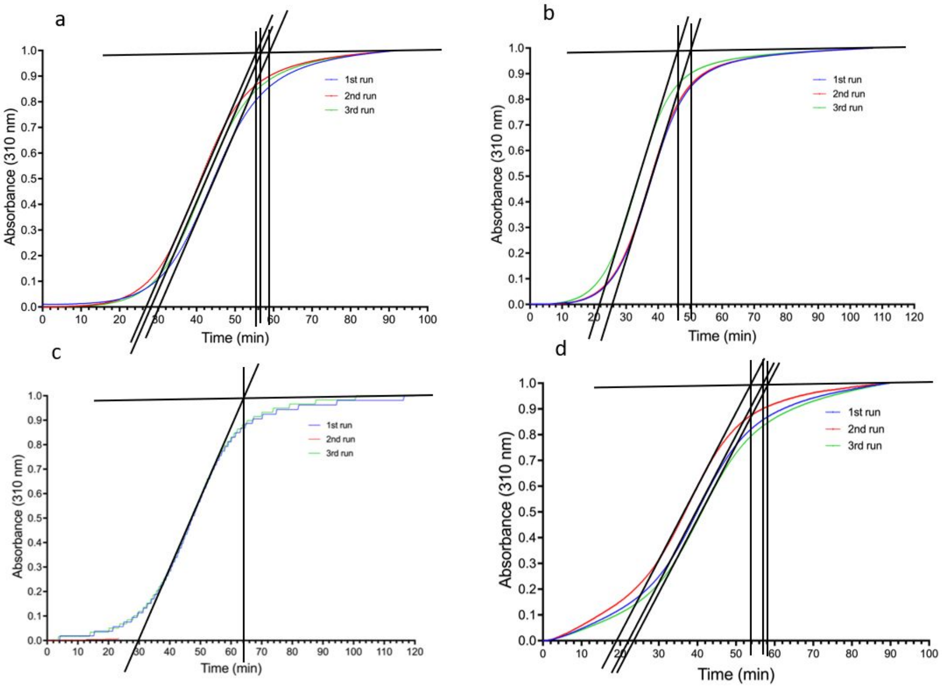


**Supplemental Figure 15:** Turbidity-time curve at 298 K +/- isoforms at 0.1 mg/mL. (a). 0.15 mg/mL collagen type I, (b). 0.15 mg/mL collagen type I with 0.1 mg/mL isoform A, (c). 0.15 mg/mL collagen type I with 0.1 mg/mL isoform B, and (d). 0.15 mg/mL collagen type I with 0.1 mg/mL isoform 0.

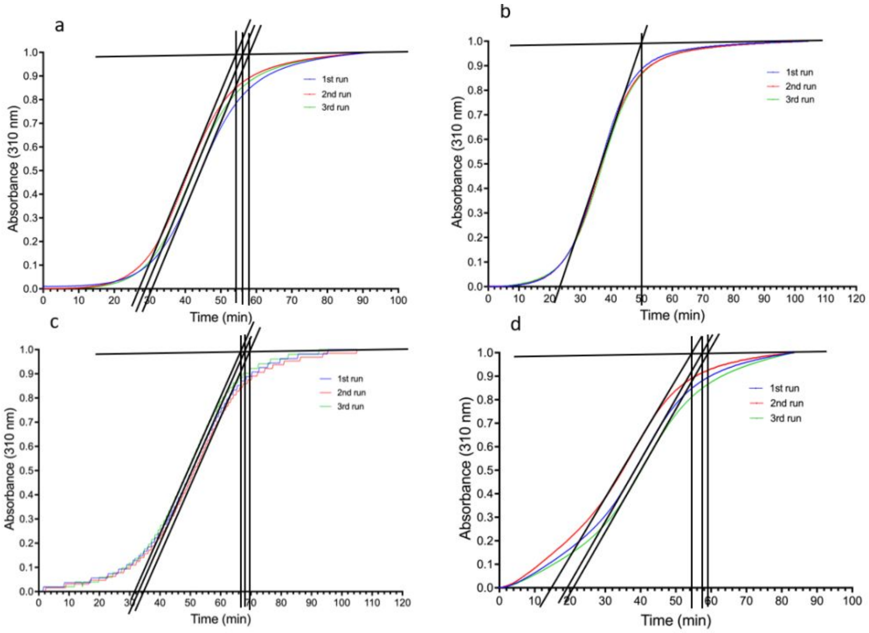


**Supplemental Figure 16:** Turbidity-time curve at 298 K +/- isoforms at 0.15 mg/mL. (a). 0.15 mg/mL collagen type I, (b). 0.15 mg/mL collagen type I with 0.15 mg/mL isoform A, (c). 0.15 mg/mL collagen type I with 0.15 mg/mL isoform B, and (d). 0.15 mg/mL collagen type I with 0.15 mg/mL isoform 0.

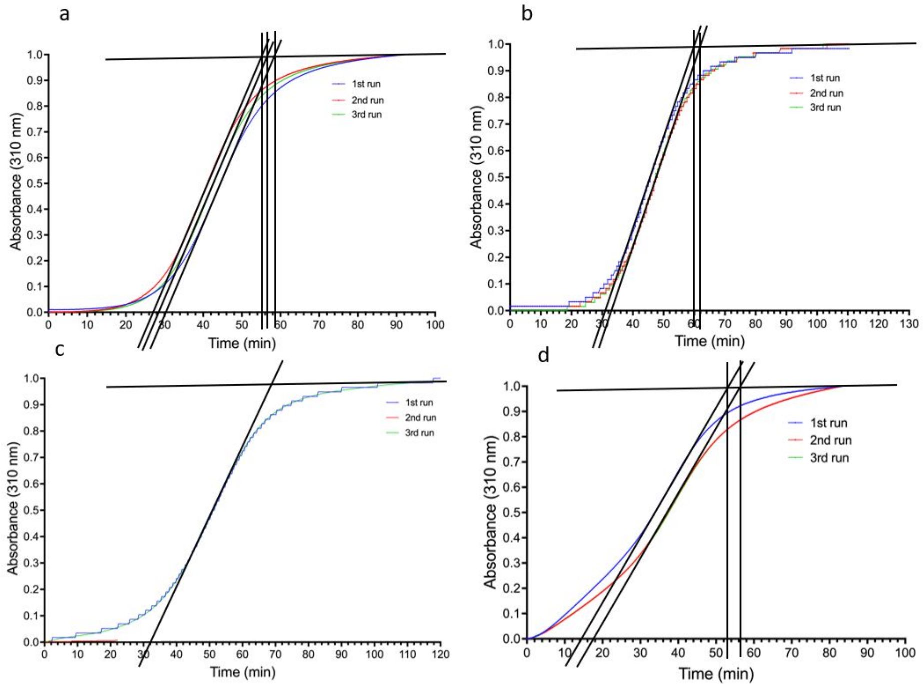


**Supplemental Figure 17:** Turbidity-time curve at 298 K +/- isoforms at 0.2 mg/mL. (a). 0.15 mg/mL collagen type I, (b). 0.15 mg/mL collagen type I with 0.2 mg/mL isoform A, (c). 0.15 mg/mL collagen type I with 0.2 mg/mL isoform B, and (d). 0.15 mg/mL collagen type I with 0.2 mg/mL isoform 0.

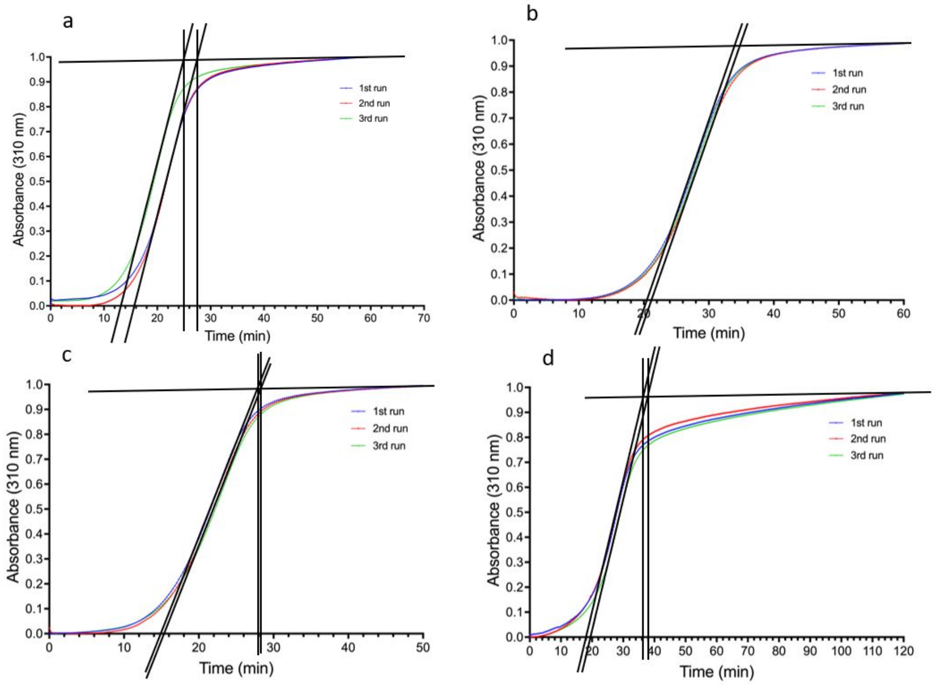


**Supplemental Figure 18:** Turbidity-time curve at 303 K +/- isoforms at 0.05 mg/mL. (a). 0.15 mg/mL collagen type I, (b). 0.15 mg/mL collagen type I with 0.05 mg/mL isoform A, (c). 0.15 mg/mL collagen type I with 0.05 mg/mL isoform B, and (d). 0.15 mg/mL collagen type I with 0.05 mg/mL isoform 0.

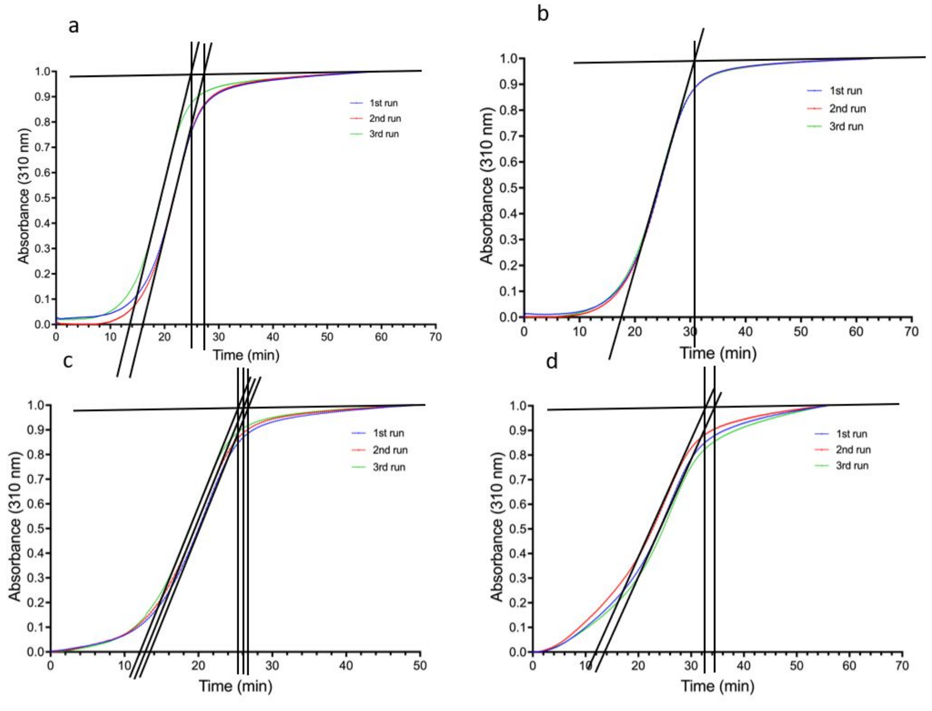


**Supplemental Figure 19:** Turbidity-time curve at 303 K +/- isoforms at 0.1 mg/mL. (a). 0.15 mg/mL collagen type I, (b). 0.15 mg/mL collagen type I with 0.1 mg/mL isoform A, (c). 0.15 mg/mL collagen type I with 0.1 mg/mL isoform B, and (d). 0.15 mg/mL collagen type I with 0.1 mg/mL isoform 0.

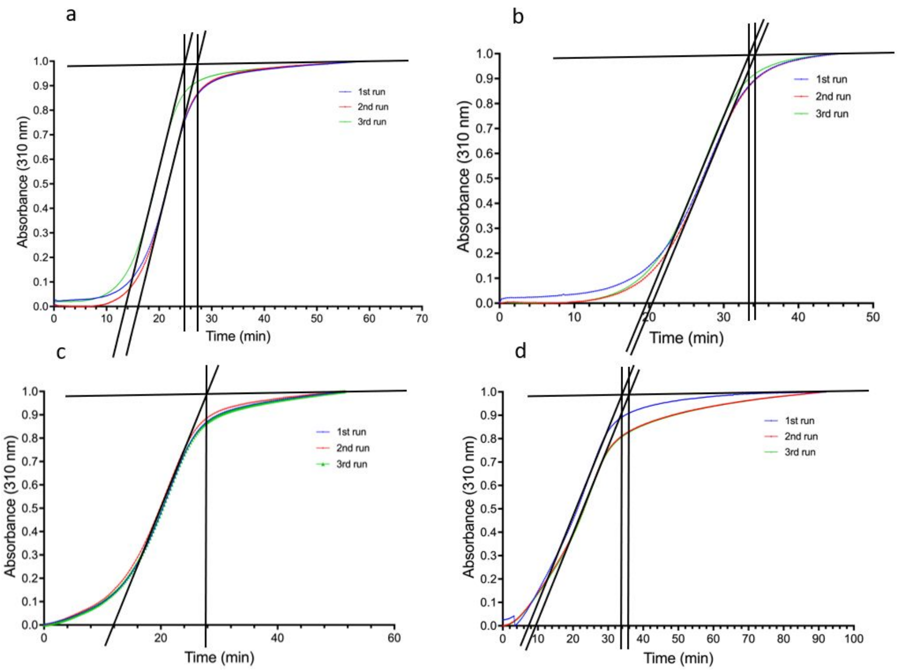


**Supplemental Figure 20:** Turbidity-time curve at 303 K +/- isoforms at 0.15 mg/mL. (a). 0.15 mg/mL collagen type I, (b). 0.15 mg/mL collagen type I with 0.15 mg/mL isoform A, (c). 0.15 mg/mL collagen type I with 0.15 mg/mL isoform B, and (d). 0.15 mg/mL collagen type I with 0.15 mg/mL isoform 0.

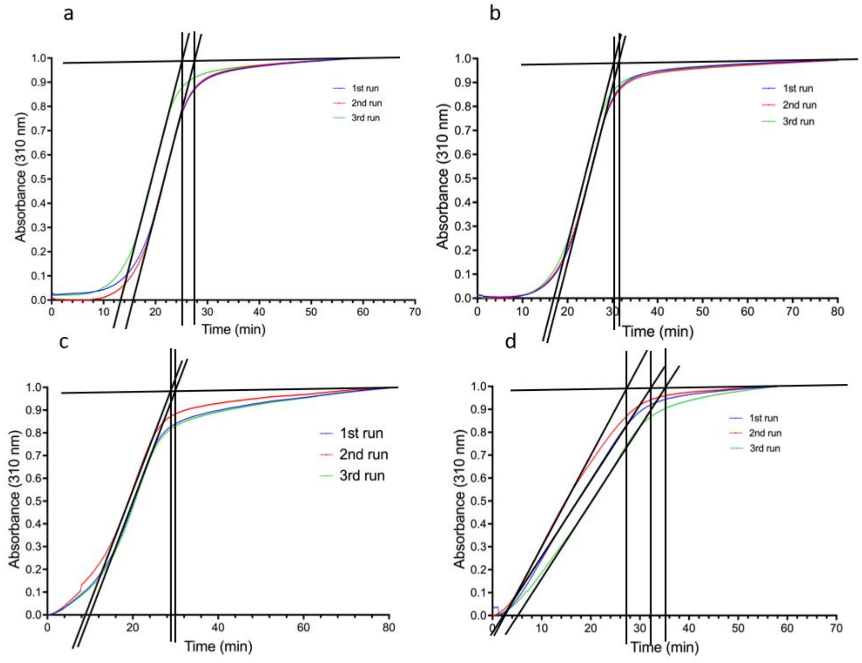


**Supplemental Figure 21:** Turbidity-time curve at 303 K +/- isoforms at 0.2 mg/mL. (a). 0.15 mg/mL collagen type I, (b). 0.15 mg/mL collagen type I with 0.2 mg/mL isoform A, (c). 0.15 mg/mL collagen type I with 0.2 mg/mL isoform B, and (d). 0.15 mg/mL collagen type I with 0.2 mg/mL isoform 0.

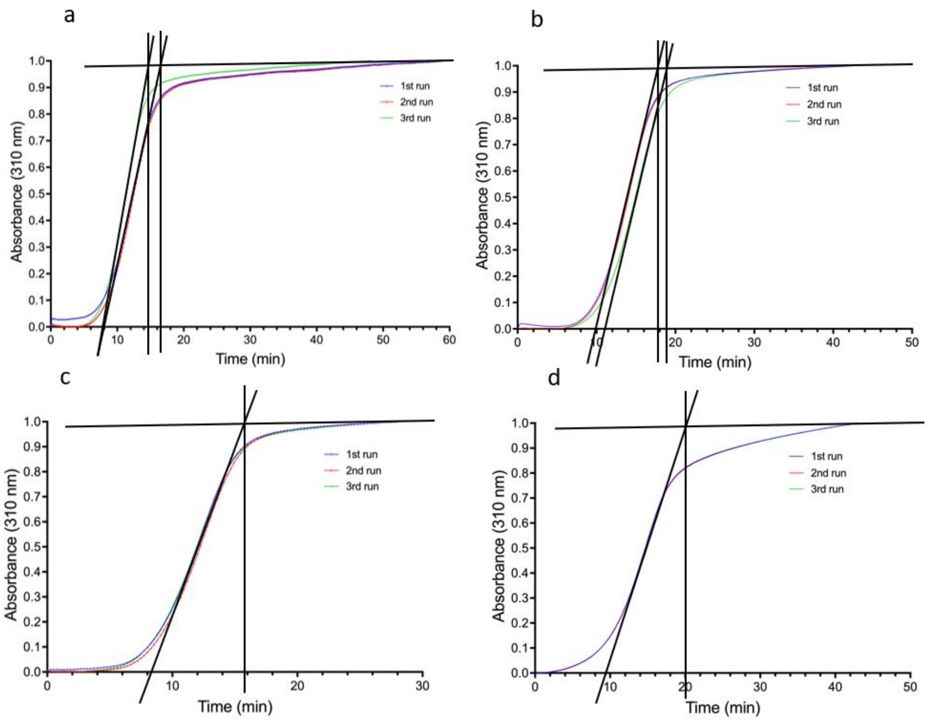


**Supplemental Figure 22:** Turbidity-time curve at 308 K +/- isoforms at 0.05 mg/mL. (a). 0.15 mg/mL collagen type I, (b). 0.15 mg/mL collagen type I with 0.05 mg/mL isoform A, (c). 0.15 mg/mL collagen type I with 0.05 mg/mL isoform B, and (d). 0.15 mg/mL collagen type I with 0.05 mg/mL of isoform 0.

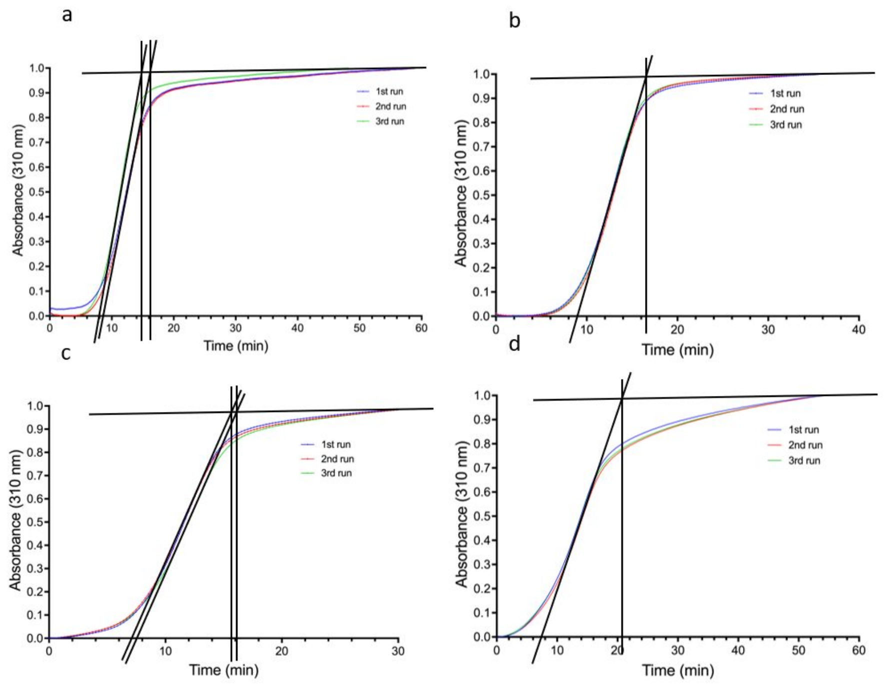


**Supplemental Figure 23:** Turbidity-time curve at 308 K +/- isoforms at 0.1 mg/mL. (a). 0.15 mg/mL collagen type I, (b). 0.15 mg/mL collagen type I with 0.1 mg/mL isoform A, (c). 0.15 mg/mL collagen type I with 0.1 mg/mL isoform B, and (d). 0.15 mg/mL collagen type I with 0.1 mg/mL isoform 0.

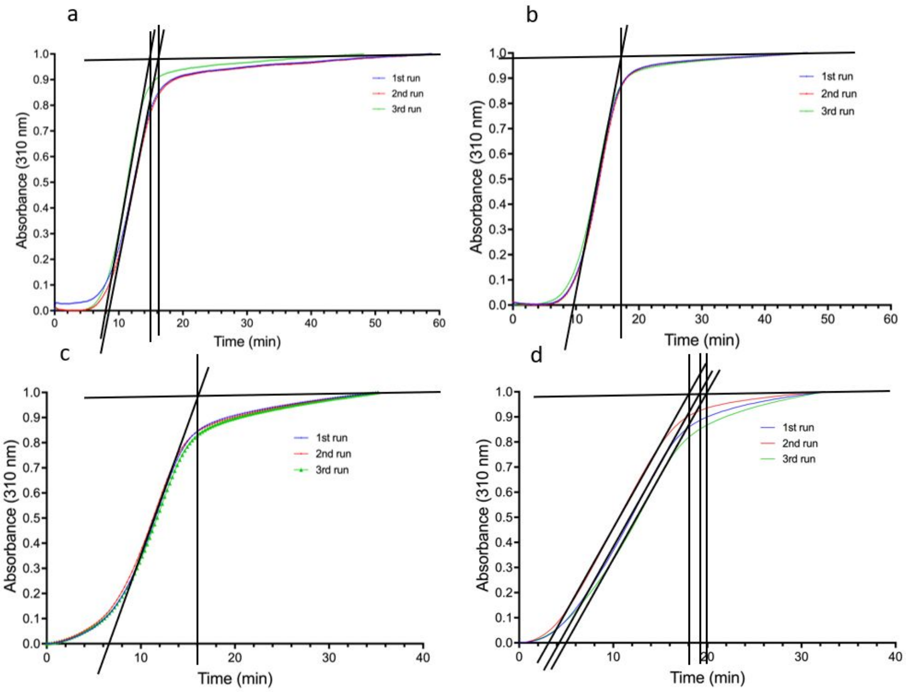


**Supplemental Figure 24:** Turbidity-time curve at 308 K +/- isoforms at 0.15 mg/mL. (a). 0.15 mg/mL collagen type I, (b). 0.15 mg/mL collagen type I with 0.15 mg/mL isoform A, (c). 0.15 mg/mL collagen type I with 0.15 mg/mL isoform B, and (d). 0.15 mg/mL collagen type I with 0.15 mg/mL isoform 0.

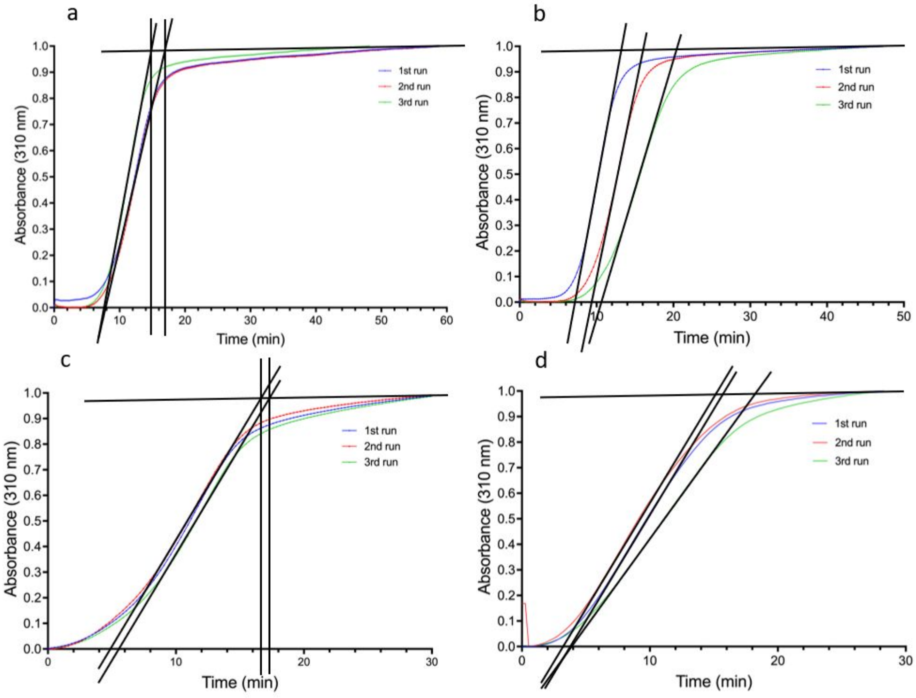


**Supplemental Figure 25:** Turbidity-time curve at 308 K +/- isoforms at 0.2 mg/mL. (a). 0.15 mg/mL collagen type I, (b). 0.15 mg/mL collagen type I with 0.2 mg/mL isoform A, (c). 0.15 mg/mL collagen type I with 0.2 mg/mL isoform B, and (d). 0.15 mg/mL collagen type I with 0.2 mg/mL isoform 0

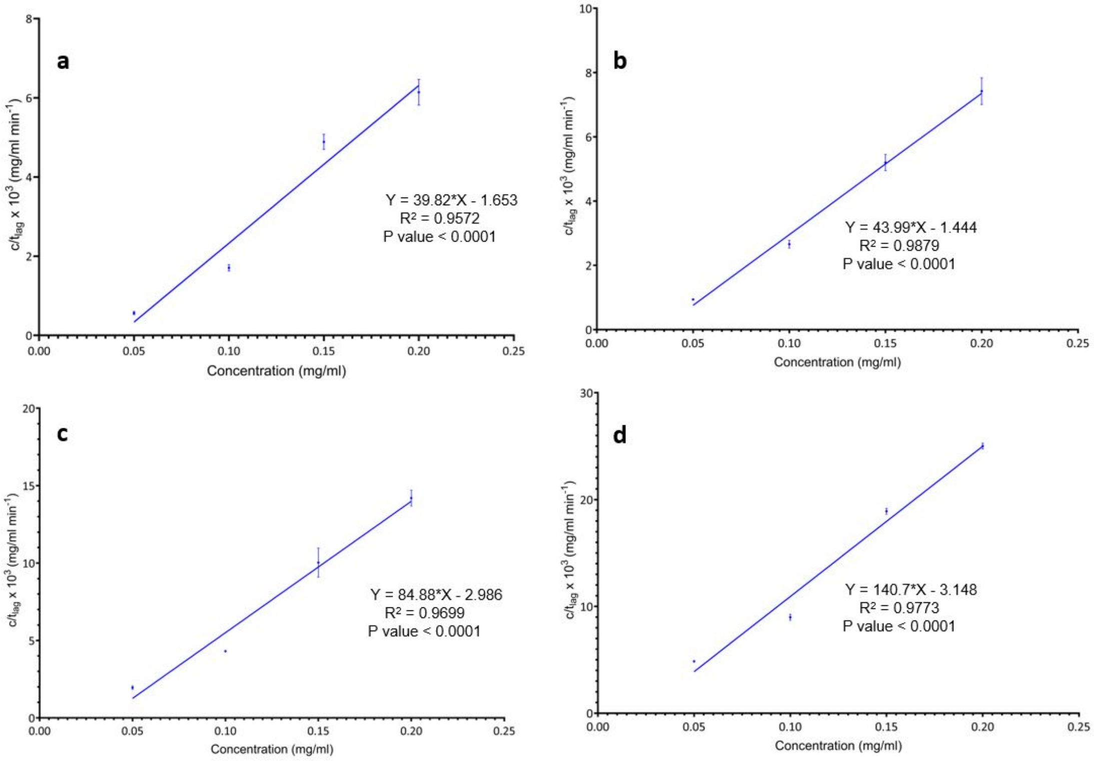


**Supplemental Figure 26:** Apparent rate constant for the control at lag phase, (a). 293 K, (b). 298 K, (c). 303 K, and (d). 308 K.

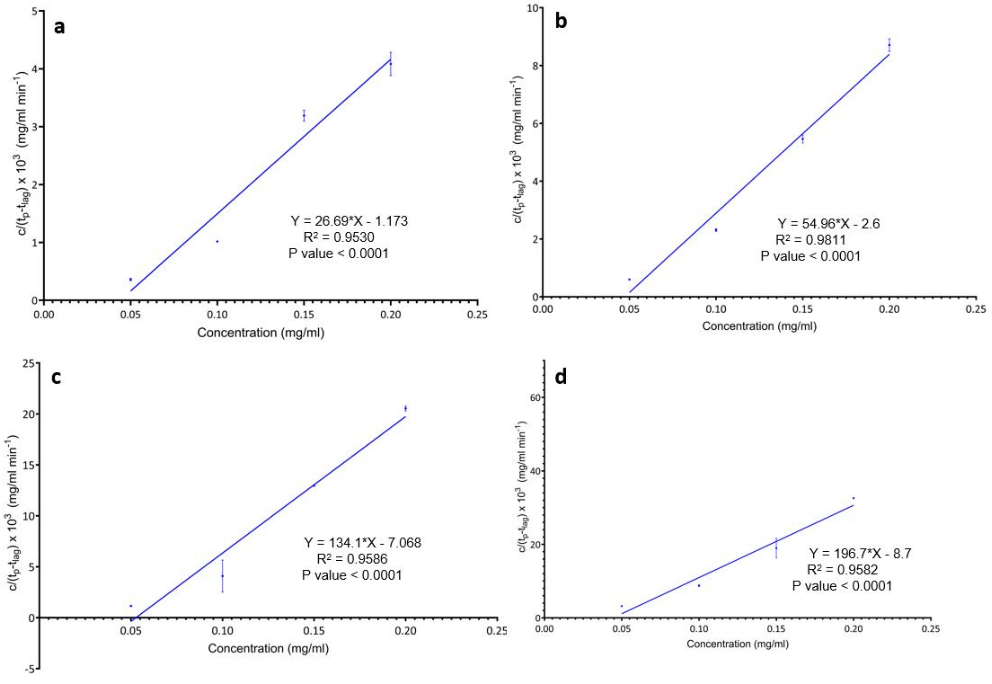


**Supplemental Figure 27:** Apparent rate constant for the control at growth phase (a). 293 K, (b). 298 K, (c). 303 K, and (d). 308 K.

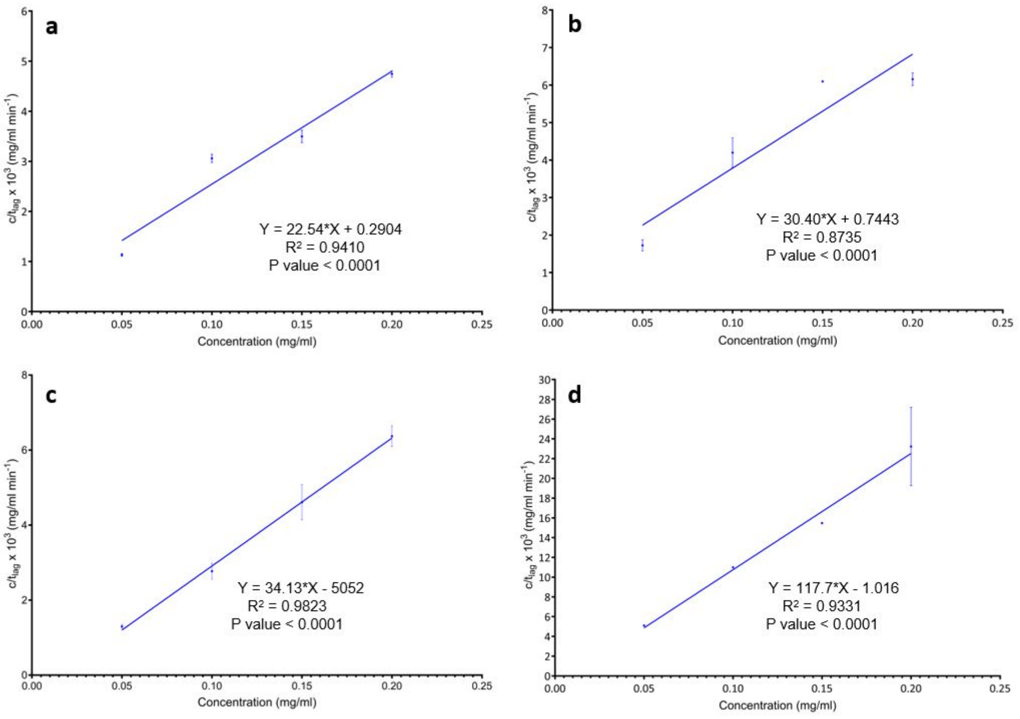


**Supplemental Figure 28:** Apparent rate constant for the isoform A at lag phase (a). 293 K, (b). 298 K, (c). 303 K, and (d). 308 K.

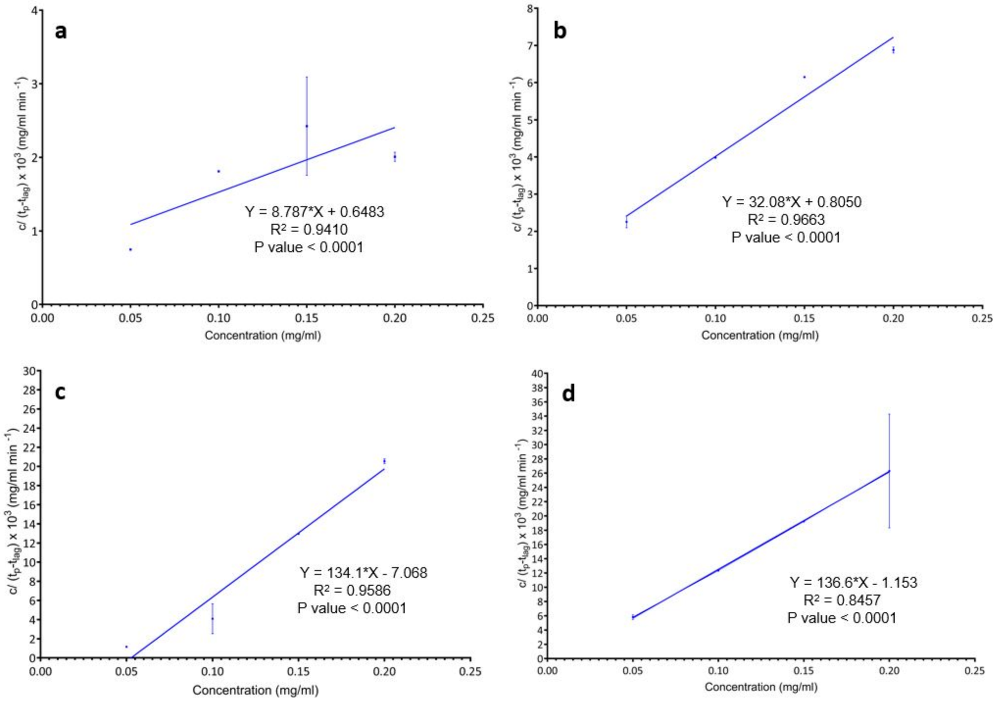


**Supplemental Figure 29:** Apparent rate constant for the isoform A at growth phase (a). 293 K, (b). 298 K, (c). 303 K, and (d). 308 K.

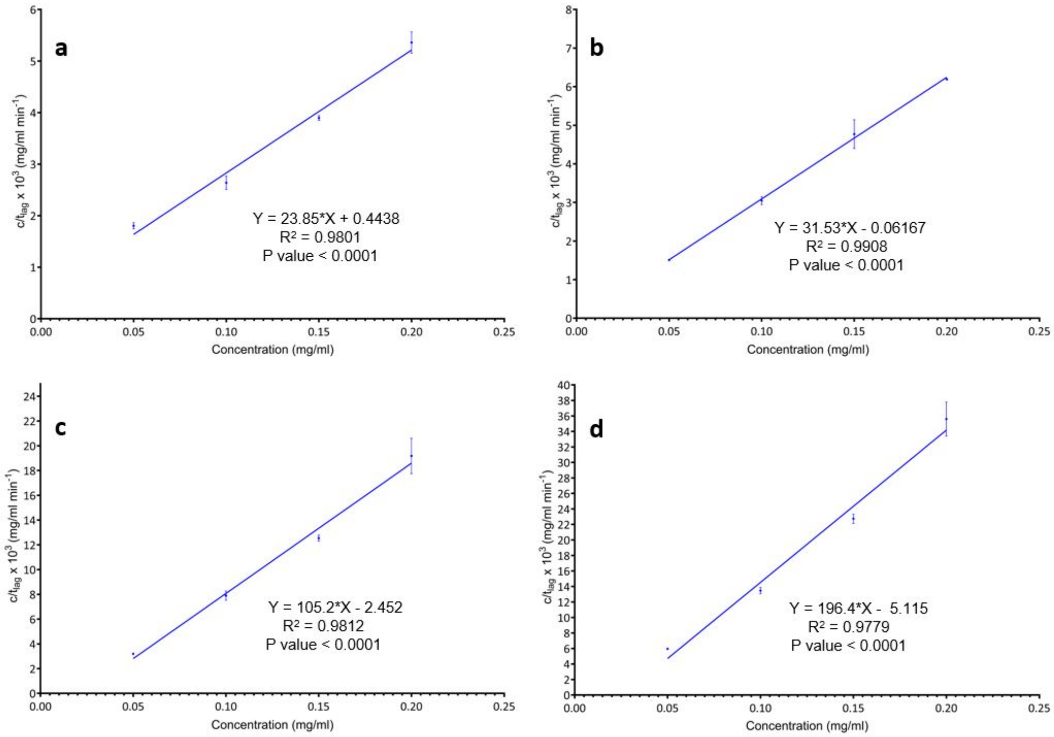


**Supplemental Figure 30:** Apparent rate constant for the isoform B at lag phase (a). 293 K, (b). 298 K, (c). 303 K, and (d). 308 K.

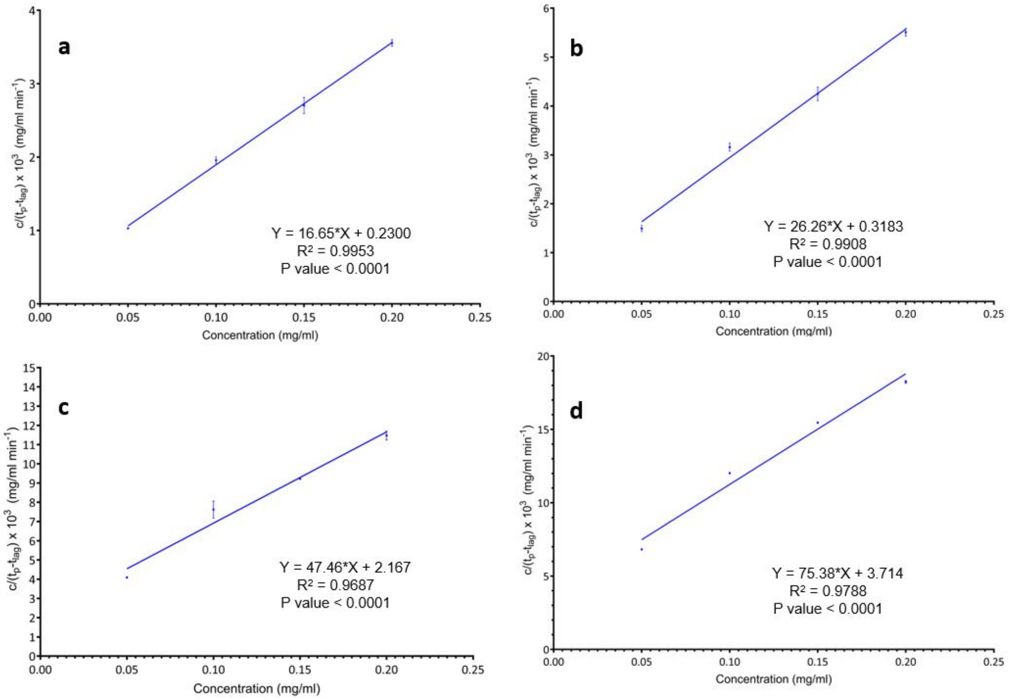


**Supplemental Figure 31:** Apparent rate constant for the isoform B at growth phase (a). 293 K, (b). 298 K, (c). 303 K, and (d). 308 K.

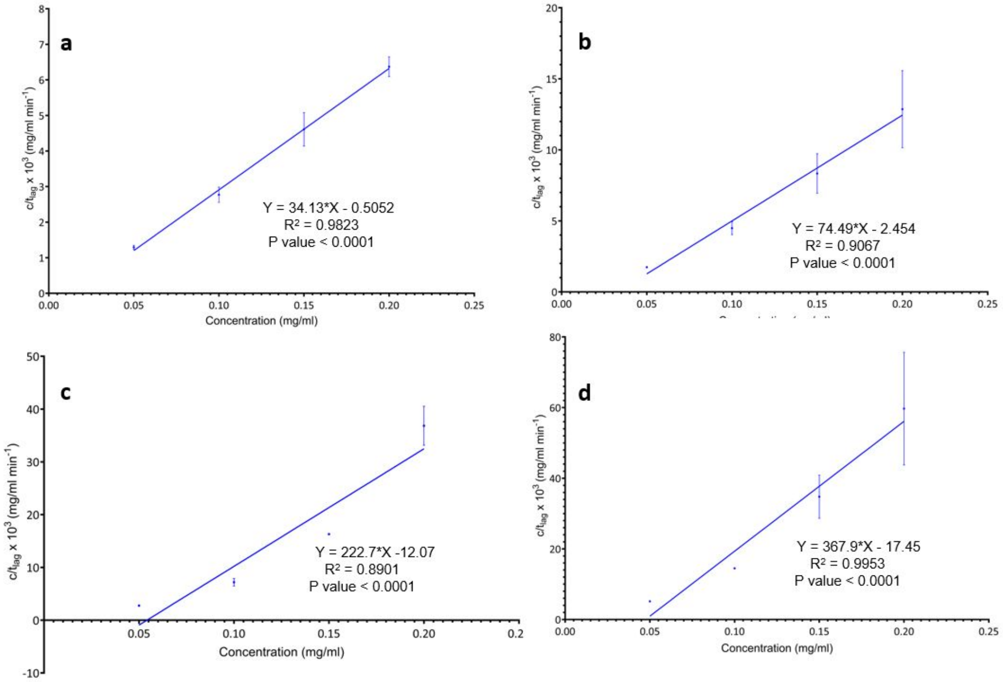


**Supplemental Figure 32:** Apparent rate constant for the isoform 0 at lag phase (a). 293 K, (b). 298 K, (c). 303 K, and (d). 308 K.

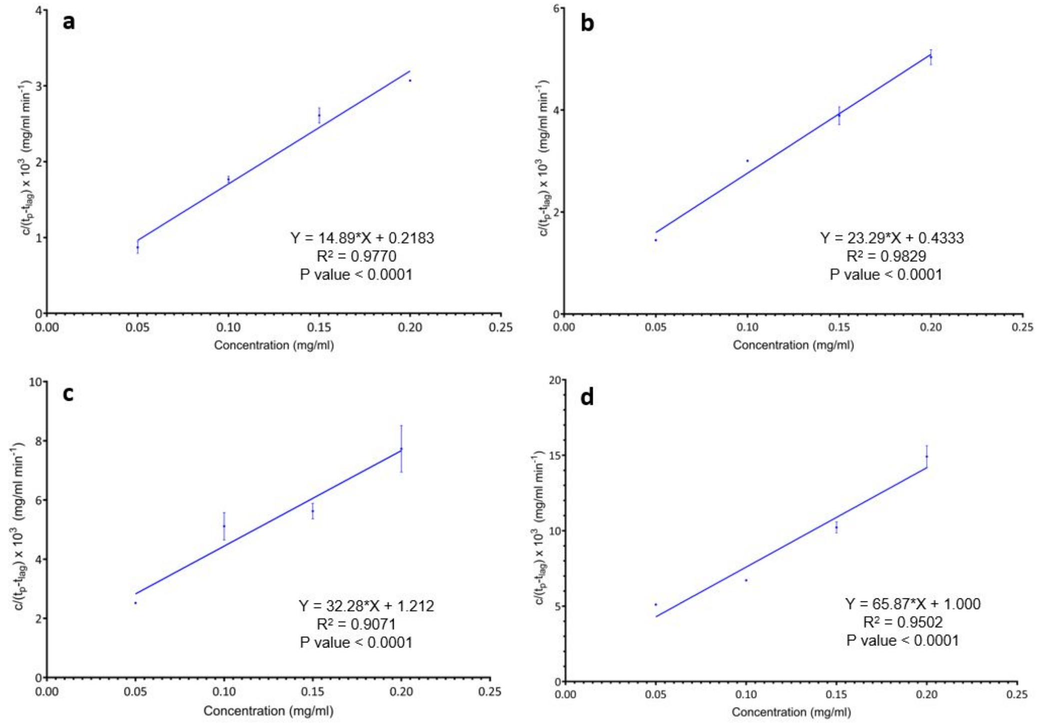


**Supplemental Figure 33:** Apparent rate constant for the isoform 0 at growth phase (a). 293 K, (b). 298 K, (c). 303 K, and (d). 308 K.

## Supplemental Tables

**Supplemental Table 1:** Summary of proteins used in this study. Exons encoding the regions of interest, theoretical pI, and percentage of acidic and basic amino acid residues are listed.

| Protein/domain | Exon | pI | Chemical nature | % acidic and basic |
|---|---|---|---|---|
| Collagen type I α-1 chain | - | 8.50 | Acidic residues | 5.1% |
| | | | Basic residues | 6.4% |
| Collagen type I α-2 chain | - | 9.99 | Acidic residues | 3.9% |
| | | | Basic residues | 7.7% |
| Variable region of isoform A | 6A | 3.46 | Acidic residues | 33.3% |
| | | | Basic residues | 2.6% |
| Variable region of isoform B | 6B | 11.25 | Acidic residues | 2% |
| | | | Basic residues | 39.2% |
| Npp | 2-5 | 8.92 | Acidic residues | 10.2% |
| | | | Basic residues | 12.5% |

**Supplemental Table 2:** Collagen type I α1 and α2 sequences. Alpha chain sequences with modified residues to hydroxyproline (**P**) and hydroxylysine (**K**) (marked bold and underlined) are shown.

| | |
|---|---|
| **Chain α1** | GPAGFAG**P**PGADGQ**P**GAKGE**P**GDTGVKGDAGP**P**GPAGPAGP**P**GPIGNVGA**P**GPKG SRGAAGP**P**GATGF**P**GAAGRVGP**P** |
| **Chain α2** | GPNGFAGPAGSAGQ**P**GA**K**GEKGTKGPKGENGIVGPTGPVGAAGPSGPNGPPGPAG SRGDGGPPGMTGF**P**GAAGRTGP**P** |

**Supplemental Table 3:** Rate constants at the lag phase (Mean +/- standard deviation)

| Temperature (K) | Control ($min^{-1}$) | Isoform 0 ($min^{-1}$) | Isoform A ($min^{-1}$) | Isoform B ($min^{-1}$) |
|---|---|---|---|---|
| **293** | 39.82 $\pm$ 2.664 | 34.13 $\pm$ 1.447 | 22.54 $\pm$ 1.785 | 23.85 $\pm$ 1.075 |
| **298** | 43.99 $\pm$ 1.537 | 74.49 $\pm$ 7.557 | 30.4 $\pm$ 3.658 | 31.53 $\pm$ 0.9607 |
| **303** | 84.88 $\pm$ 4.732 | 222.7 $\pm$ 24.75 | 34.13 $\pm$ 1.447 | 105.2 $\pm$ 4.602 |
| **308** | 140.7 $\pm$ 6.784 | 367.9 $\pm$ 45.38 | 117.7 $\pm$ 9.968 | 196.4 $\pm$ 9.342 |

**Supplemental Table 4:** Rate constants at the growth phase (Mean +/- standard deviation)

| Temperature (K) | Control ($min^{-1}$) | Isoform 0 ($min^{-1}$) | Isoform A ($min^{-1}$) | Isoform B ($min^{-1}$) |
|---|---|---|---|---|
| **293** | 26.69 ± 1.874 | 14.89 ± 0.7215 | 8.787 ± 2.632 | 16.65 ± 0.3613 |
| **298** | 54.96 ± 2.414 | 23.29 ± 0.9729 | 32.02 ± 1.896 | 26.26 ± 0.8597 |
| **303** | 134.1 ± 8.811 | 32.28 ± 3.267 | 134.1 ± 8.811 | 47.46 ± 2.699 |
| **308** | 196.7 ± 13.00 | 65.87 ± 4.770 | 136.6 ± 18.46 | 75.38 ± 3.508 |

**Supplemental Table 5:** Gromacs molecular mechanics Poisson-Boltzmann Surface Area (gmx_mmpbsa) results. (ΔBOND- Change in bond energy, ΔANGLE- Change in angle energy, ΔDIHED- Change in dihedral energy, ΔUB- Change in Urey-Bradley energy, ΔIMP- Change in improper-dihedral energy, ΔCMAP- Change in correction map energy, ΔVDWAALS- Change in Van der Waals energy, ΔEEL- Change in electrostatic energy, Δ1-4 VDW- Change in 1-4 Van der Waals energy, Δ1-4 EEL- Change in 1-4 electrostatic energy, ΔEGB- Change in Generalized Born energy, ΔESURF- Change in surface area energy, ΔGGAS- Change in gas-phase energy, ΔGSOLV- Change in solvation energy)

| | **Isoform A** | | **Isoform B** | | **Isoform 0** | |
|---|---|---|---|---|---|---|
| | **Energy (kcal/mol)** | **SEM** | **Energy kcal/mol)** | **SEM** | **Energy (kcal/mol)** | **SEM** |
| **ΔBOND** | 0 | 0 | 0 | 0 | 0 | 0 |
| **ΔANGLE** | 0 | 0 | 0 | 0 | 0 | 0 |
| **ΔDIHED** | 0 | 0 | 0 | 0 | 0 | 0 |
| **ΔUB** | 0 | 0 | 0 | 0 | 0 | 0 |
| **ΔIMP** | 0 | 0 | 0 | 0 | 0 | 0 |
| **ΔCMAP** | 0 | 0 | 0 | 0 | 0 | 0 |
| **ΔVDWAALS** | -28.91 | 0.05 | -10.21 | 0.12 | -35.12 | 0.23 |
| **ΔEEL** | -354.09 | 0.31 | 374.58 | 2.64 | -323.58 | 1.66 |
| **Δ1-4 VDW** | 0 | 0 | 0 | 0 | 0 | 0 |
| **Δ1-4 EEL** | 0 | 0 | 0 | 0 | 0 | 0 |
| **ΔEGB** | 369.34 | 0.29 | -361.57 | 2.51 | 344.97 | 1.78 |
| **ΔESURF** | -4.71 | 0.01 | -1.23 | 0.01 | -4.38 | 0.03 |
| **ΔGGAS** | -383 | 0.31 | 364.37 | 2.52 | -358.71 | 1.88 |
| **ΔGSOLV** | 364.63 | 0.28 | -362.8 | 2.52 | 340.59 | 1.75 |
| **ΔTOTAL** | -18.37 | 0.07 | 1.57 | 0.03 | -18.11 | 0.14 |

**Supplemental Table 6:** Parameters for molecular dynamics simulation

| ions.mdp | npt.mdp | nvt.mdp | md.mdp |
|---|---|---|---|
| Integrator = steep<br><br>emtol = 1000.0<br>emstep = 0.01<br>nsteps = 50000<br><br><br>nstlist = 1<br>cutoff-scheme = Verlet<br>ns_type = grid<br>rlist = 1.0<br>coulombtype = cutoff<br>rcoulomb = 1.0<br>rvdw = 1.0<br>pbc = xyz | define = -DPOSRES<br>integrator = md<br>nsteps = 200000<br>dt = 0.002<br>nstxout = 500<br>nstvout = 500<br>nstenergy = 500<br>nstcomm = 100<br>nstlog = 500<br>continuation = yes<br>constraint_algorithm = lincs constraints = h-bonds<br>lincs_iter = 1<br>lincs_order = 4<br>cutoff-scheme = Verlet<br>ns_type = grid<br>rlist = 1.2<br>nstlist = 10<br>rcoulomb = 1<br>rcoulomb-switch = 0<br>rvdw = 1<br>coulombtype = PME<br>pme_order = 6<br>fourierspacing = 0.12<br>vdw-type = Cut-off<br>DispCorr = EnerPres<br>tcoupl = V-rescale<br>tc-grps = Protein Non-Protein tau_t = 0.1 0.1<br>ref_t = 310 310<br>pcoupl = Parrinello-Rahman pcoupltype = isotropic<br>tau_p = 1<br>ref_p = 1<br>compressibility = 4.5e-5<br>refcoord_scaling = com<br>pbc = xyz<br>gen_vel = no | define = -DPOSRES<br>integrator = md<br>nsteps = 50000<br>dt= 0.002<br>nstxout = 500<br>nstvout = 500<br>nstenergy = 500<br>nstcomm = 100<br>nstlog = 500<br>continuation = no<br>constraint_algorithm = lincs<br>constraints = h-bonds<br>lincs_iter = 1<br>lincs_order = 4<br>cutoff-scheme = Verlet<br>ns_type = grid<br>rlist = 1.2<br>nstlist = 10<br>rcoulomb = 1<br>rcoulomb-switch = 0<br>rvdw = 1<br>coulombtype = PME<br>pme_order = 6<br>fourierspacing = 0.12<br>vdw-type = Cut-off<br>DispCorr = EnerPres<br>tcoupl = V-rescale<br>tc-grps = Protein Non-Protein<br>tau_t = 0.1 0.1<br>ref_t = 310 310<br>pcoupl = no<br>pbc = xyz<br>gen_vel = yes<br>gen_temp = 310<br>gen_seed = -1 | integrator = md<br>nsteps = 50000000<br>dt = 0.002<br>nstxout = 500<br>nstvout = 500<br>nstenergy = 500<br>nstcomm = 100<br>nstlog = 500<br>continuation = yes<br>constraint_algorithm = lincs<br>constraints = h-bonds<br>lincs_iter = 1<br>lincs_order = 4<br>cutoff-scheme = Verlet<br>ns_type = grid<br>rlist = 1.2<br>nstlist = 20<br>rcoulomb = 1.2<br>rvdw = 1.2<br>rvdw-switch = 1.0<br>coulombtype = PME<br>pme_order = 6<br>fourierspacing = 0.12<br>vdw-type = Cut-off<br>vdw-modifier = force-switch<br>DispCorr = EnerPres<br>tcoupl = V-rescale<br>tc-grps = Protein Non-Protein<br>tau_t = 0.1 0.1<br>ref_t = “” “”<br>pcoupl = Parrinello-Rahman<br>pcoupltype = isotropic<br>tau_p = 2<br>ref_p = 1<br>compressibility = 4.5e-5<br>pbc = xyz<br>gen_vel = no |

**Supplemental Table 7:** Parameters for Gromacs molecular mechanics Poisson-Boltzmann Surface Area (gmx_mmpbsa)

| General namelist variables &general | (AMBER) Generalized-Born namelist variables &gb |
|---|---|
| sys_name = "" | igb = 5 |
| startframe = “” | intdiel = 1.0 |
| endframe = “” | extdiel = 78.5 |
| interval = 1 | saltcon = 0.0 |
| forcefields = "oldff/leaprc.ff99SB,leaprc.gaff" | surften = 0.0072 |
| ions_parameters = 1 | surfoff = 0.0 |
| PBRadii = 3 | molsurf = 0 |
| temperature = 310 | msoffset = 0.0 |
| qh_entropy = 0 | probe = 1.4 |
| interaction_entropy = 0 | ifqnt = 0 |
| ie_segment = 25 | qm_theory = "" |
| c2_entropy = 0 | qm_residues = "" |
| assign_chainID = 0 | qmcharge_com = 0 |
| exp_ki = 0.0 | qmcharge_lig = 0 |
| full_traj = 0 | qmcharge_rec = 0 |
| gmx_path = "" | qmcut = 9999.0 |
| keep_files = 2 | scfconv = 1e-08 |
| netcdf = 0 | peptide_corr = 0 |
| solvated_trajectory = 1 | writepdb = 1 |
| verbose = 1 | verbosity = 0 |
| | alpb = 0 |
| | arad_method = 1 |

**Abbreviations**

ΔBOND- Change in bond energy, ΔANGLE- Change in angle energy, ΔDIHED- Change in dihedral energy, ΔUB- Change in Urey-Bradley energy, ΔIMP- Change in improper-dihedral energy, ΔCMAP- Change in correction map energy, ΔVDWAALS- Change in Van der Waals energy, ΔEEL- Change in electrostatic energy, Δ1-4 VDW- Change in 1-4 Van der Waals energy, Δ1-4 EEL- Change in 1-4 electrostatic energy, ΔEGB- Change in Generalized Born energy, ΔESURF- Change in surface area energy, ΔGGAS- Change in gas-phase energy, ΔGSOLV- Change in solvation energy.
